# Prevalence of nocturnal and isolated nocturnal hypertension in population-based studies: a systematic review and meta-analysis

**DOI:** 10.3389/fcvm.2026.1883404

**Published:** 2026-10-28

**Authors:** Víctor Juan Vera-Ponce, Jhosmer Ballena-Caicedo, Lupita Ana Maria Valladolid-Sandoval, Fiorella E. Zuzunaga-Montoya

**Affiliations:** Facultad de Medicina (FAMED), Universidad Nacional Toribio Rodríguez de Mendoza de Amazonas (UNTRM), Chachapoyas, Amazonas, Peru

**Keywords:** blood pressure, ambulatory blood pressure monitoring, cardiovascular diseases, circadian rhythm, epidemiology, hypertension, prevalence

## Abstract

**Background:**

Nocturnal hypertension (NH) and isolated nocturnal hypertension (INH) require nighttime blood pressure assessment, but published prevalence estimates differ in sampling, treatment eligibility, and ambulatory blood pressure monitoring (ABPM) protocols.

**Objective:**

This study aimed to summarize NH and study-defined INH proportions in population/community samples.

**Methods:**

Four databases were searched through 17 March 2026. Logit random-effects models used restricted maximum likelihood and modified Hartung–Knapp confidence intervals (CIs). Risk of bias was assessed using the nine-item Joanna Briggs Institute (JBI) 2017 checklist. Clinical/occupational and circadian-pattern reports were described individually rather than pooled.

**Results:**

Nine NH estimates from four sources included 8,282 participants and 3,331 cases. Pooled prevalence was 42.5% (95% CI, 33.2%–52.3%; approximate 95% prediction interval, 17.2%–72.3%; I^2^ = 97.6%). Four INH estimates included 3,659 participants and 657 cases; the descriptive pooled proportion was 17.3% (95% CI, 10.4%–27.5%; prediction interval, 3.6%–54.0%; I^2^ = 91.5%). INH denominators differed by treatment eligibility; a sensitivity analysis using reported non-treated denominators yielded 19.6% (95% CI, 11.4%–31.6%). Certainty was judged very low. A sensitivity requiring both original nighttime recording-quality criteria could not be estimated from the published aggregate data.

**Conclusions:**

NH and INH were common in the population-based and community samples studied. These findings provide reference estimates for planning population-based studies and evaluating targeted detection strategies using ABPM.

**Systematic Review Registration:**

https://www.crd.york.ac.uk/PROSPERO/view/CRD420251233166, identifier PROSPERO CRD420251233166.

## Introduction

In 2024, approximately 1.4 billion adults aged 30–79 years were living with hypertension worldwide, corresponding to 33% of the population in that age range; approximately 600 million were unaware of their diagnosis, and only 320 million had controlled blood pressure (1). In the Region of the Americas, age-standardized prevalence among adults aged 30–79 years was 35.4% [95% uncertainty interval (UI), 33.3%–37.6%] in 2019, with higher prevalence among men than women; in Peru, the comparable regional estimate was 20.7% (95% UI, 18.2%–23.3%), although the WHO national profile estimated 3.2 million Peruvian adults aged 30–79 years with hypertension (2, 3). This burden remains substantial: in the Americas, prevalence increased by 7.6% between 1990 and 2019 and has remained nearly stagnant since 2010, while WHO describes a global increase from 650 million hypertensive adults in 1990 to 1.4 billion in 2024 (1, 2).

Nocturnal hypertension (NH) cannot be identified from clinic blood pressure measurements alone. The reference method for assessing it is 24-h ambulatory blood pressure monitoring (ABPM), using validated devices and classifying the nocturnal period through sleep diaries, narrow time intervals, or, in more specialized designs, actigraphy or polysomnography (4). Recent consensus documents define NH as mean nighttime systolic blood pressure ≥120 mmHg and/or diastolic blood pressure ≥70 mmHg and isolated nocturnal hypertension (INH) as the same nighttime threshold in the presence of daytime blood pressure <135/85 mmHg (4, 5). The clinical uncertainty is not whether nocturnal blood pressure matters, but how frequent these phenotypes are when measured using heterogeneous ABPM protocols, in populations with different baseline risk and under variable definitions of the sleep period.

Prognostic evidence has progressively shifted attention from office blood pressure to ambulatory blood pressure monitoring, particularly the nighttime period. In the Spanish Ambulatory Blood Pressure Registry, which included 59,124 primary care patients with a median follow-up of 9.7 years, nighttime systolic pressure was the most informative measure for all-cause and cardiovascular mortality compared with clinic systolic pressure (6). In the Japan Ambulatory Blood Pressure Monitoring Prospective (JAMP) study, including 6,359 patients at high cardiovascular risk, nighttime systolic pressure and the riser pattern were associated with cardiovascular events and, especially, heart failure (7). In parallel, population-based and clinical primary studies have reported variable prevalences of isolated nocturnal hypertension, ranging from the Chinese JingNing study to the Jackson Heart Study and Korean cohorts; however, these data have remained scattered across regions, populations, diagnostic thresholds, and types of nocturnal phenotypes (8–10).

The central epidemiological question is how often elevated nighttime pressure occurs in community samples, rather than whether nighttime pressure predicts cardiovascular outcomes. Interpreting prevalence requires identifying who could be included in the denominator, whether antihypertensive treatment affected classification, and how valid nighttime recordings were defined. A pooled result cannot replace these study-specific descriptions, particularly when only a few sources are available.

The primary objective was to summarize NH prevalence in population/community samples using 24-h ABPM and the nighttime 120/70-mmHg threshold. The secondary quantitative objective was to summarize study-defined INH proportions and conduct treatment-denominator sensitivity analyses. Clinical, occupational, high-risk, alternative-threshold, and circadian-pattern reports were summarized as contextual evidence. The review is registered in PROSPERO (CRD420251233166), and reporting follows PRISMA 2020 (11).

## Methods

### Protocol registration and scope

The review was registered in PROSPERO under identifier CRD420251233166 with the title Global Prevalence of Nocturnal Hypertension: A Systematic Review and Meta-Analysis. INH and dipping phenotypes were listed as additional outcomes, when reported, in the available protocol. Supplementary Table S1 provides the PRISMA checklist. Changes to eligibility, statistical methods, and risk-of-bias assessment are summarized in Supplementary Table S2.

Eligibility was broadened during full-text review to include clinical, occupational, and high-risk samples. Reassessment of previously excluded titles and abstracts under the expanded criteria was not documented, and no repeat screening was undertaken during revision. The quantitative synthesis was therefore restricted to population/community studies; additional reports were retained as non-exhaustive contextual evidence.

### Eligibility and outcome definitions

The population question used the condition–context–population framework: NH or INH, measurement by 24-h ABPM, and general-population or community-based recruitment. Eligible designs were cross-sectional studies and cohort-derived prevalence analyses with an identified ABPM device and sufficient outcome-specific counts. Referral-only, occupational, hospitalized, institutionalized, pregnancy, dialysis, exclusively pediatric, and acutely decompensated samples were excluded from the population meta-analyses. Reviews, consensus documents, editorials, letters, and case series with fewer than 30 participants were not quantitative sources. Community substudies restricted by treatment, age, or additional measurements were retained as explicitly restricted samples rather than as unrestricted national populations.

NH was defined as mean nighttime systolic pressure ≥120 mmHg and/or diastolic pressure ≥70 mmHg. The Jackson Heart Study used >120 or >70 mmHg. INH additionally required daytime pressure <135/85 mmHg, subject to each source's treatment rules. JingNing included eight adolescents; adult-specific event counts were unavailable, so its published denominator was retained and examined by whole-study omission. Alternative thresholds and systolic-only definitions were not combined with the primary outcome (8–10).

The protocol required at least 70% successful nighttime readings and at least 10 valid nighttime measurements. Reports with lower study-specific minima were retained when the outcome was extractable. Supplementary Table S6a records each source's nighttime minimum and whether the reported validity percentage concerns nighttime or the full 24 h. A strict-protocol sensitivity required an extractable numerator and denominator meeting both original nighttime criteria.

### Information sources and search strategies

PubMed/MEDLINE, Embase, Scopus, and Web of Science Core Collection were searched without language or publication-date restrictions, with a reported final search date of 17 March 2026. The database-specific strategies are presented in Supplementary Table S3a. Gray literature, trial registries, and conference abstracts were not searched.

The searches focused on NH and nighttime blood pressure; dedicated non-dipping and reverse/riser terms were not consistently included across databases. No supplementary search was performed for these phenotypes. Their results were therefore retained only as non-exhaustive contextual observations, without a circadian-pattern meta-analysis.

### Study selection and data extraction

Three reviewers independently screened titles/abstracts and full texts, with decisions masked until comparison and a pilot of 50 records. Disagreements were resolved by consensus or a fourth reviewer. Two reviewers extracted data in parallel, and a third checked a sample of forms. Supplementary Table S3b identifies the role of each full-text report in the revised synthesis.

Extracted variables included source, recruitment frame, population restrictions, age and sex, antihypertensive treatment, ABPM device and frequency, night definition, recording-quality requirements, outcome definition, cases, and denominator. Explicit counts were preferred. Total NH was derived from mutually exclusive INH and day–night hypertension categories when available. Rounded percentages were not used to create primary population events. Approximate contextual counts, including the TEleMonitoring of blood Pressure in Local phARmacies (TEMPLAR) project, remain explicitly identified. No individual ABPM records, adult-only event counts, or unreported treatment histories were imputed.

A study denotes the underlying investigation; a source denotes the original publication or collaborative dataset; a cohort denotes a distinct participant sample; and an analytical component denotes one outcome-specific row in a synthesis. The International Database on Ambulatory blood pressure in relation to Cardiovascular Outcomes (IDACO) contributes six non-overlapping components, including one that combines four European Project on Genes in Hypertension (EPOGH) cohorts. Its 189-person JingNing component was omitted to avoid overlap with Li/JingNing, leaving 6,357 of the publication's 6,546 participants in the NH dataset (12).

#### Treatment denominators for INH

The descriptive INH synthesis summarizes the study-defined phenotype as a proportion of each publication's analytical sample. It is not labeled prevalence among all untreated adults. JingNing retained 71 treated participants with day–night hypertension but excluded 25 treated participants with other pressure patterns; its INH numerator was untreated within the 677-person denominator. KoGES required no history of antihypertensive medication for INH but used a 1,734-person denominator containing 527 participants on treatment. The Jackson Heart Study and Rhee excluded current antihypertensive treatment (8–10, 13).

A *post hoc* denominator sensitivity analysis used 606 participants for JingNing (677−71), 1,207 for KoGES (1,734−527), and the original untreated denominators for the Jackson Heart Study and Rhee, without changing the INH numerators. Current treatment and treatment history could not be fully harmonized. Original and sensitivity denominators are reported in Supplementary Table S6b.

#### Risk-of-bias assessment

Risk of bias was assessed using the nine-item JBI Critical Appraisal Checklist for Studies Reporting Prevalence Data, 2017 version, retaining its original questions and response categories: Yes, No, Unclear, and Not applicable (14, 15). Three authors performed the risk-of-bias assessment for each of the five population sources. Assistance from ChatGPT 5.5 (free version, developed by OpenAI; https://chatgpt.com/) was limited to organizing instructions for applying the checklist and checking the study content relevant to its items. The three authors verified the AI-assisted content directly against the available primary study reports and made item-level judgments; a fourth author resolved discrepancies. All final judgments were made by the authors, not by the language model. Item-level judgments and their justifications are presented in Supplementary Tables S7a, S7b.

No numerical score, overall quality category, or D5–D8 exclusion rule was used. Applicability to the review's population question was assessed separately. For Q8, the prevalence precision reported by the primary study differed from the intervals calculated in this review. The assessment informed interpretation and certainty rather than study exclusion.

#### Statistical synthesis and uncertainty

For each extractable outcome, prevalence was calculated as the number of cases divided by the stated analytical denominator. Individual 95% confidence intervals (CIs) were calculated by the Wilson method. Population estimates were synthesized on the logit scale using inverse-variance random-effects models. Between-component variance, *τ*^2^, was estimated by restricted maximum likelihood (REML). The pooled confidence interval used modified Hartung–Knapp inference with *k* − 1 degrees of freedom and q* = max(1,*q*), where *q* is the weighted residual mean square. The adjusted variance was q*/*Σw*, with *w* = 1/(*v* + *τ*^2^). Point estimates and limits were transformed back using the inverse-logit function (16, 17).

The pooled proportions describe an average analytical component on the logit scale, not a population-weighted prevalence across countries. Heterogeneity was assessed using Cochran's *Q*, *Q*-based *I*^2^, *τ*^2^, and approximate prediction intervals (PIs). *I*^2^ was calculated as max[0,(*Q* − (*k* − 1))/*Q*] × 100%. Prediction intervals used *t* with *k* − 2 degrees of freedom and √(*τ*^2^ + *V*), where *V* is the adjusted variance of the pooled logit; these intervals remain approximate in few sources.

The protocol specified Freeman–Tukey transformation with DerSimonian–Laird estimation. The previous revision used a logit model with DerSimonian–Laird and normal confidence intervals; the timing of that change relative to inspection of interim results was not documented. REML with modified Hartung–Knapp inference was adopted in response to the second-round concern about uncertainty in few-study settings. DerSimonian–Laird/normal and Freeman–Tukey/DerSimonian–Laird models were retained as sensitivity analyses, with harmonic-mean inverse transformation for Freeman–Tukey (18).

Sensitivity analyses compared models, omitted one analytical component or source at a time, excluded JingNing, and examined INH treatment denominators. A two-stage NH analysis first pooled the six IDACO components using REML and a modified Hartung–Knapp variance, then combined the resulting estimate and adjusted variance with Li, Jackson Heart Study, and Rhee. The second stage treats the estimated first-stage variance as fixed. The strict nocturnal-quality sensitivity could not be estimated when no extractable denominator met the documented original criteria.

Clinical, occupational, high-risk, systolic-only, and circadian-pattern reports were summarized individually by sampling frame and outcome definition, without a combined prevalence. Meta-regression was not retained because the evidence was sparse and region, setting, and case mix could not be separated reliably.

The revised statistical analyses were performed in Python using NumPy, SciPy, and Pandas.

### Reporting bias and certainty

No formal funnel-plot asymmetry test, Egger regression, or trim-and-fill analysis is retained. The population syntheses include four original sources per outcome, and combining distinct clinical and community frames solely to increase *k* would not yield a coherent assessment. Search coverage and missing outcome data were considered qualitatively. Reporting bias remains uncertain, and the absence of a formal test is not evidence of its absence.

Certainty was assessed using an approach informed by Grading of Recommendations Assessment, Development and Evaluation (GRADE) for prevalence, rather than a separately validated prevalence-specific GRADE instrument. Appropriate population cross-sectional or cohort-derived designs were not automatically downgraded solely because they were observational. This starting point follows the event-frequency/prognosis analogy while recognizing methodological limitations of its transfer to prevalence (19, 20). Risk of bias, inconsistency, indirectness, imprecision, and missing-result concerns were described separately for each outcome (Supplementary Table S8).

The revised judgments do not use a summed JBI score, the retired core-domain rule, or the previous *post hoc* 10-percentage-point confidence-interval cutoff. Serious risk-of-bias concerns reflect the documented selection and coverage problems; very serious inconsistency reflects marked differences between estimates and broad prediction intervals; and serious indirectness concerns extrapolation from restricted age, treatment, or measurement subgroups to an unrestricted adult population. No decision-relevant precision threshold was prespecified, so confidence-interval widths are reported without an additional threshold-based downgrade. This does not imply adequate precision. Both summaries were judged to have very low certainty in the stated domains.

## Results

### Selection and revised analytical disposition

The reported searches identified 4,790 records: 875 from Scopus, 1,923 from Embase, 876 from PubMed/MEDLINE, and 1,116 from the Web of Science Core Collection. After removal of 1,313 duplicates, 3,477 records underwent title/abstract screening and 3,454 were excluded. Twenty-three reports were assessed in full text; the initial review retained 17 original reports and excluded 6.

Five of the 17 retained source publications contribute to the revised population syntheses. The other 12 are presented as contextual or non-extractable evidence. Figure 1 shows the selection flow, and Supplementary Table S3b gives the disposition of all 23 full-text reports.

**Figure 1:**
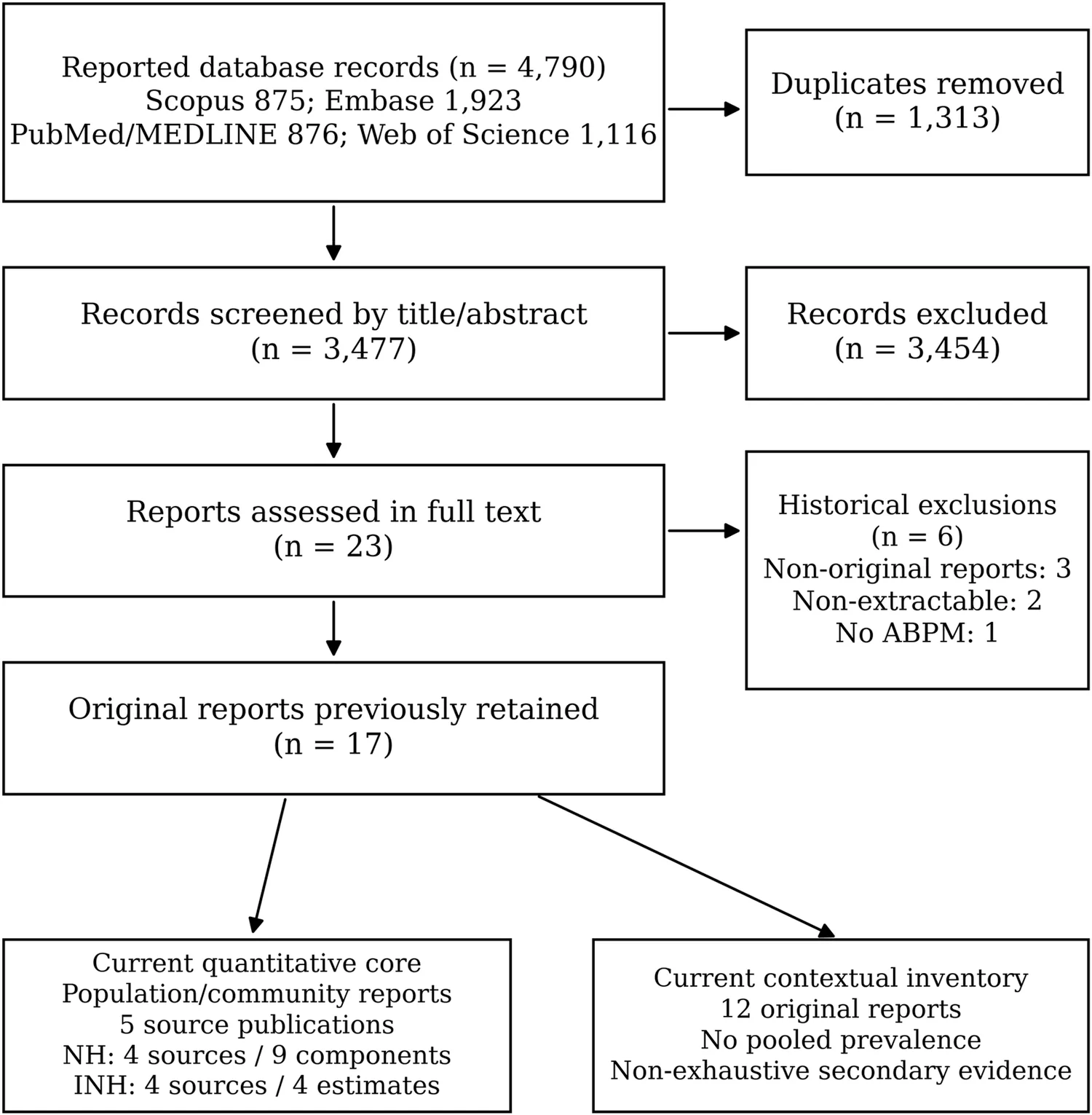
Study-selection flow and disposition of reports. Five source publications contributed to the population meta-analyses; 12 additional reports were retained as contextual evidence.

### Population sources and comparability

NH synthesis includes IDACO, JingNing, Jackson Heart Study, and Rhee/Korea; INH synthesis includes JingNing, Jackson Heart Study, Rhee/Korea, and KoGES (8–10, 12, 13). These represent five unique source publications. Outcome-specific sample sizes overlap and are not additive. Characteristics are presented in Table 1, and individual counts are summarized in Supplementary Tables S4a, S4b.

**Table 1:** Characteristics of the five source publications contributing to the population syntheses.

Source	Recruitment/setting	Analytical *N*	Age/sex	Treatment eligibility	ABPM/nighttime quality	Outcome and interpretation
Melgarejo/IDACO 2017 (12)	Population cohorts; Denmark, Japan, Belgium, Uruguay, EPOGH (four European cohorts), Venezuela	6,546 in publication; 6,357 used	Ages 40–79 years; mean 57.8 years; 44.9% women	Treated and untreated	Cohort-specific devices and timing; ≥5 nighttime readings	NH; six analytical components
Li/JingNing 2007 (8)	Rural population; China	677	Mean 47.6 years; 53.6% women; eight adolescents	71 treated day–night hypertensive participants retained; 25 other treated participants excluded	Spacelabs 90207; 22:00–04:00; ≥5 nighttime readings	NH and INH. Denominator is neither adult-only nor exclusively untreated
Ogedegbe/JHS 2013 (9)	Community cohort; United States	425	African-American adults; approximately 54 years; approximately 62% women	No current antihypertensive medication	Spacelabs 90207; sleep diary; ≥54 total readings/≥75% over 24 h	NH and INH. Voluntary ABPM substudy; threshold >120 or >70 rather than ≥
Rhee/Korea 2021 (10)	Community sample in five cities; South Korea	823	Ages 20–70; mean 47.5 years; 67.0% women	No current antihypertensive medication	Mobil-O-Graph; 00:00–06:00; >70% overall; ≥7 nighttime readings	NH and INH. Random-digit invitation supplemented by relatives/friends
Kim/KoGES 2022 (13)	Community-derived ABPM/MRI subset; South Korea	1,734	Mean 59.9 years; 54.0% women	527 current medication users in denominator; INH requires no treatment history	Mobil-O-Graph; 23:00–06:00; >70% overall; ≥7 nighttime readings	INH only. The main proportion uses all 1,734; sensitivity uses 1,207

NH, nocturnal hypertension; INH, isolated nocturnal hypertension; ABPM, ambulatory blood pressure monitoring; MRI, magnetic resonance imaging.

Participant totals overlap between NH and INH.

The IDACO publication describes 6,546 participants, of whom 6,357 are included in this extraction after removing JingNing. EPOGH aggregates four original cohorts into a single component. Other samples include a rural study with phenotype-dependent treatment exclusions and eight adolescents, an untreated African-American volunteer ABPM subset, an untreated Korean sample including invited relatives or friends, and a Korean ABPM/MRI substudy with a treatment-history restriction in the INH definition. These features limit equivalence to an unrestricted adult population, notwithstanding their community origins.

### Risk of bias and certainty

The JBI assessment identified concerns about selection into ABPM substudies, analytical coverage, and non-response. The Jackson Heart Study used a volunteer ABPM subset, Rhee included relatives and friends in recruitment, and JingNing's treated-participant exclusions depended on the pressure phenotype. Li, Ogedegbe, and Kim did not report prevalence-specific confidence intervals and were rated No for Q8. Table 2 and Figure 2 present the judgments; Supplementary Table S7b provides the justifications. Certainty was very low for both outcomes (Table 3).

**Figure 2:**
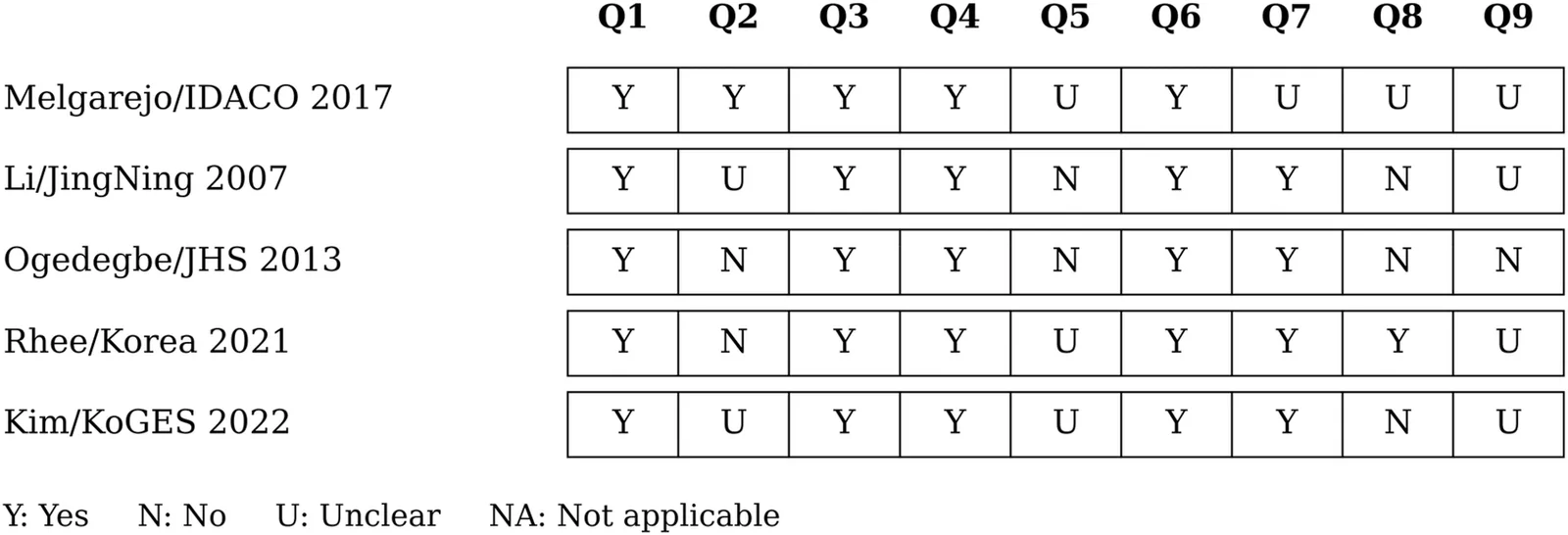
Item-level JBI 2017 appraisal of the five population source publications. Y = Yes; N = No; U = Unclear. Item definitions are given in Table 2, and justifications are provided in Supplementary Table S7b.

**Table 2:** JBI 2017 item-level appraisal and separate applicability assessment.

Source	Q1	Q2	Q3	Q4	Q5	Q6	Q7	Q8	Q9	Applicability, assessed separately
Melgarejo/IDACO 2017 (12)	Y	Y	Y	Y	U	Y	U	U	U	Restricted communities and ages 40–79 years; six extracted analytical components, including four EPOGH cohorts within one row. Not a worldwide probability sample
Li/JingNing 2007 (8)	Y	U	Y	Y	N	Y	Y	N	U	Rural sample including eight adolescents and phenotype-dependent retention of treated participants; neither an unrestricted adult nor an exclusively untreated denominator
Ogedegbe/Jackson Heart Study 2013 (9)	Y	N	Y	Y	N	Y	Y	N	N	African-American adults without current antihypertensive treatment who volunteered for ABPM
Rhee/Korea 2021 (10)	Y	N	Y	Y	U	Y	Y	Y	U	Urban participants without current treatment, recruited by a mixture of random telephone invitation and relatives/friends; not necessarily all Korean adults.
Kim/KoGES 2022 (13)	Y	U	Y	Y	U	Y	Y	N	U	ABPM/MRI community subset; 527 of 1,734 on medication, while INH requires no medication history. Current treatment and treatment history are not equivalent

Y = Yes; N = No; U = Unclear; NA = Not applicable. Q1, sampling frame; Q2, sampling method; Q3, sample size; Q4, population and setting; Q5, analytical coverage; Q6, condition identification; Q7, measurement consistency; Q8, statistical analysis; Q9, response and non-response (14).

Justifications are provided in Supplementary Table S7b. Applicability was assessed separately; no summed score was used.

**Table 3:** Summary of findings for the two retained descriptive population syntheses.

Outcome/target	Evidence size	Pooled %, 95% CI	Approximate 95% PI, %	Certainty	Principal reasons
NH in retained population/community samples	9 components; 4 sources; *N* = 8,282; 3,331 cases	42.5 (33.2–52.3)	17.2–72.3	Very low	Selection/coverage concerns; extreme unexplained heterogeneity; age, treatment, and recording-quality restrictions; IDACO extraction verification incomplete
Study-defined INH proportion in retained samples	4 estimates; 4 sources; *N* = 3,659; 657 cases	17.3 (10.4–27.5)	3.6–54.0	Very low	Selection/coverage concerns; extreme unexplained heterogeneity; inconsistent treatment rules and analytical denominators. Not untreated-population prevalence

Certainty was assessed using a GRADE-informed approach. Domain-specific judgments and reasons are presented in Supplementary Table S8.

### Population quantitative results

NH analysis included nine analytical components from four sources, with 8,282 participants and 3,331 cases. REML with the modified Hartung–Knapp inference yielded 42.5% (95% CI, 33.2%–52.3%), with *Q*-based *I*^2^ = 97.6%, *τ*^2^ = 0.257, and an approximate 95% prediction interval of 17.2%–72.3%. Individual proportions ranged from 24.7% to 63.6% (Figure 3; Table 4 and Supplementary Table S4a).

**Figure 3:**
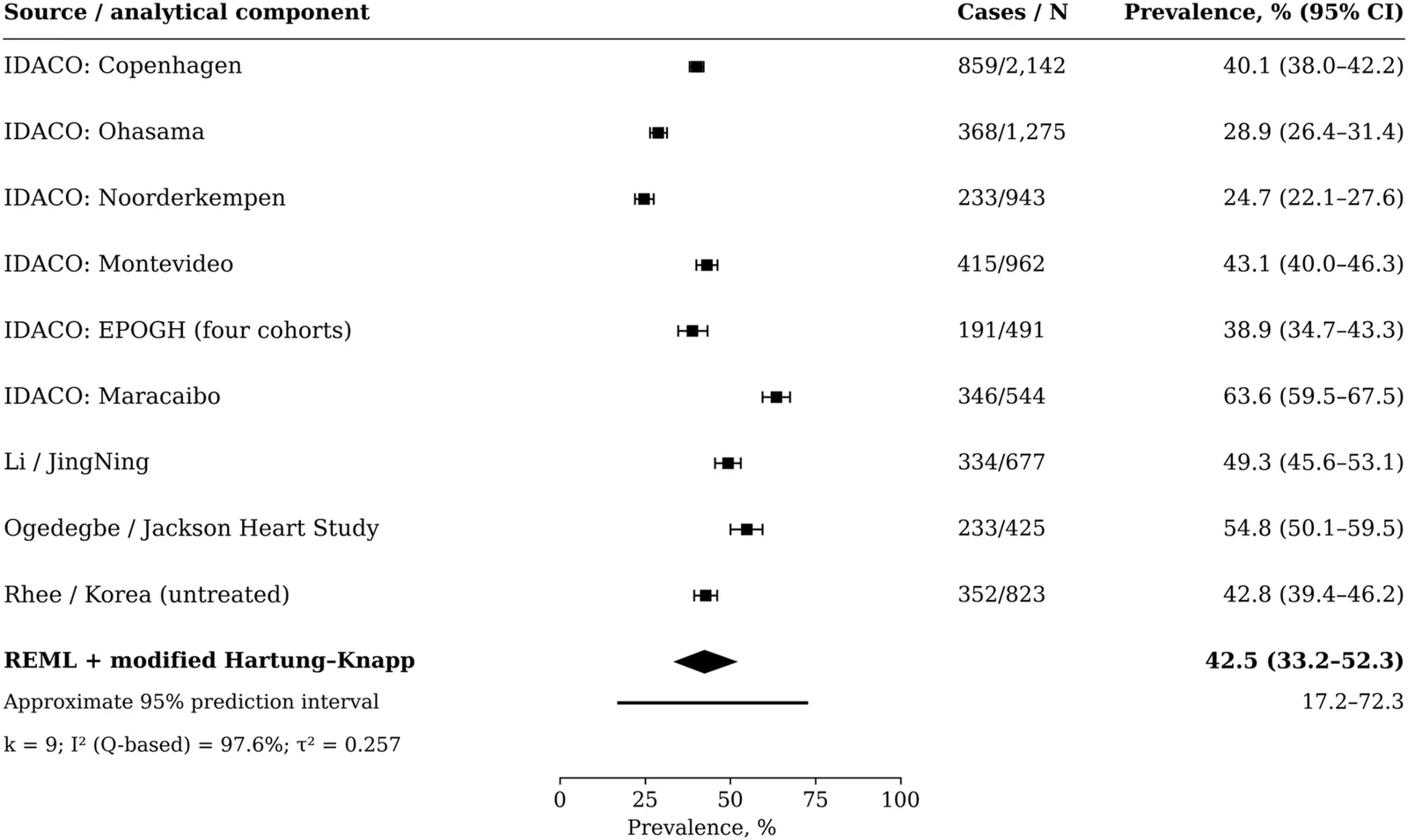
NH prevalence in nine analytical components from four population sources. EPOGH combines four cohorts into a single analytical component. Individual intervals are Wilson intervals; the pooled model uses REML and the modified Hartung–Knapp inference. Squares have uniform size; the lower line is the approximate prediction interval.

**Table 4:** Population meta-analysis results: logit–REML with modified Hartung–Knapp confidence intervals.

Outcome	*k*	Sources	*N*	Cases	Pooled %, 95% CI	*I^2^*, %	*τ*^2^ logit	Approximate 95% PI, %
NH	9	4	8,282	3,331	42.5 (33.2–52.3)	97.6	0.257	17.2–72.3
Study-defined INH	4	4	3,659	657	17.3 (10.4–27.5)	91.5	0.126	3.6–54.0

*k* counts analytical components, not publications. Confidence intervals use *t*(*k* − 1) and q* = max(1,*q*). Prediction intervals use *t*(*k* − 2) and the sum of *τ*^2^ and the adjusted pooled-logit variance; they are approximate. *I*^2^ is *Q*-based throughout this manuscript. The study-defined INH average is not a harmonized estimate among all untreated adults.

The four study-defined INH estimates comprised 3,659 participants and 657 cases. Their descriptive pooled proportion was 17.3% (95% CI, 10.4%–27.5%), with *I*^2^ = 91.5%, *τ*^2^ = 0.126, and an approximate prediction interval of 3.6%–54.0% (Figure 4; Table 4 and Supplementary Table S4b). This is an average of published analytical-sample proportions, not an estimate for a uniformly untreated population. Using the reported non-treated denominators in a *post hoc* sensitivity analysis retained 657 cases but reduced the total denominator to 3,061, yielding 19.6% (95% CI, 11.4%–31.6%; prediction interval, 3.6%–61.5%).

**Figure 4:**
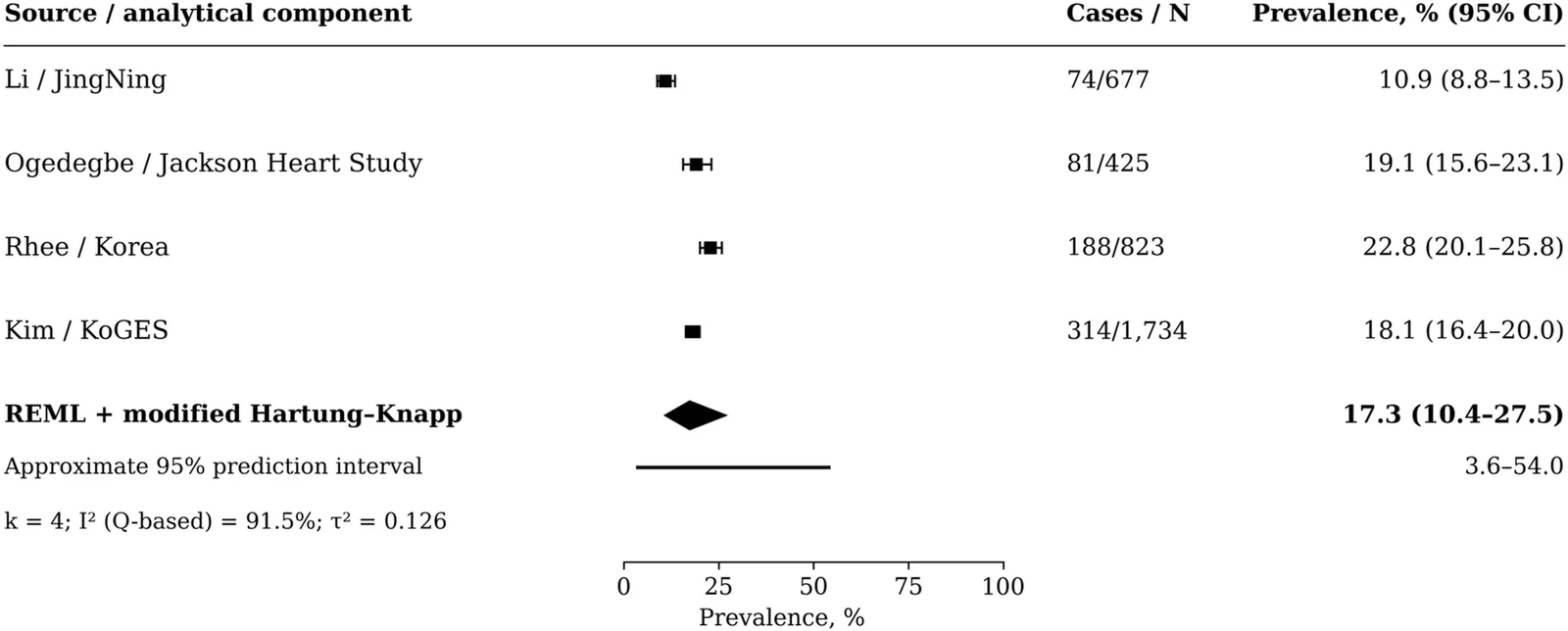
Study-defined INH proportions using original analytical denominators. Individual intervals are Wilson intervals; the diamond is the modified Hartung–Knapp interval, and the lower line is the approximate prediction interval. Squares have uniform size, not study weights. Treatment eligibility differs across sources (Supplementary Table S6b).

### Sensitivity and recording-quality audit

With identical counts, the DerSimonian–Laird/normal inference gave NH 42.5% (95% CI, 35.4%–49.9%) and INH 17.4% (95% CI, 13.3%–22.3%). The Freeman–Tukey/DerSimonian–Laird inference gave 42.6% (95% CI, 35.4%–50.0%) and 17.5% (95% CI, 13.0%–22.5%), respectively. Thus, the point estimates remained similar, but REML with the modified Hartung–Knapp inference produced wider confidence intervals and greater estimated uncertainty than the DerSimonian–Laird/normal inference. Model comparisons are reported in Table 5 and Supplementary Table S5.

**Table 5:** Sensitivity analyses and their interpretation.

Analysis	Result, % (95% CI unless stated)	Interpretation
DerSimonian-Laird (DL) with normal intervals	NH 42.5% (35.4%–49.9%); INH 17.4% (13.3%–22.3%)	Same analytical counts; normal intervals are narrower
Freeman–Tukey + DL	NH 42.6% (35.4%–50.0%); INH 17.5% (13.0%–22.5%)	Transformation comparison, not reproduction of all original eligibility rules
NH, two-stage source weighting	47.3 (37.1–57.8); PI 22.2%–73.8%	Four sources; first-stage IDACO variance is estimated and treated conditionally in stage two
Omit the entire IDACO source	48.8% (34.3%–63.5%); three sources	Assesses the influence of IDACO on the pooled estimate
Leave one analytical estimate out	NH 39.9%–45.0%; INH 15.7%–19.9%	Range of pooled point estimates, not a confidence interval or demonstration of transportability
Omit JingNing entirely	NH 41.6% (31.3%–52.8%); INH 19.9% (14.2%–27.3%)	Examines the source with eight adolescents and phenotype-dependent treatment selection; other sample restrictions remain
INH, reported non-treated denominators	19.6 (11.4–31.6); *N* = 3,061; 657 cases	*Post hoc* contrast; does not equate current medication with treatment history or correct selection
Original nighttime quality criteria	Not estimable	No extractable denominator with documented ≥70% nighttime success and ≥10 valid nighttime readings

The two-stage source-level NH analysis yielded 47.3% (95% CI, 37.1%–57.8%; approximate prediction interval, 22.2%–73.8%). Omitting IDACO left three sources and yielded 48.8% (95% CI, 34.3%–63.5%). Leave-one-component-out estimates ranged from 39.9% to 45.0% for NH and 15.7% to 19.9% for INH. Omitting JingNing gave NH 41.6% (95% CI, 31.3%–52.8%) and INH 19.9% (95% CI, 14.2%–27.3%). These analyses assess influence on the pooled estimate, not transportability across populations.

No source provided an extractable numerator and denominator that demonstrably meets both original nighttime-quality requirements. IDACO and JingNing accepted at least five nighttime readings, Rhee and KoGES accepted at least seven, and Jackson Heart Study specified an overall 24-h completeness criterion. The strict-protocol pooled sensitivity could therefore not be estimated (Supplementary Table S6a). This reflects insufficient information to establish compliance rather than evidence that every recording was invalid.

### Contextual evidence

Individual NH proportions in the contextual non-population reports ranged from 34.8% to 79.2% across tertiary-care patients, cardiometabolic outpatients, university employees, and a high-risk clinical cohort. NH and INH results are presented in Supplementary Tables S4c, S4d. Alternative-threshold, systolic-only, and circadian-pattern results are presented in Supplementary Tables S4e–S4h without pooled estimates.

## Discussion

### Principal findings and interpretation

This review brings together the available evidence on NH and INH in population-based and community samples, providing a quantitative synthesis of their frequency. The pooled prevalence of NH was 42.5% (95% CI, 33.2%–52.3%), while the pooled proportion of study-defined INH was 17.3%. The approximate prediction interval for NH (17.2%–72.3%) illustrates the variation across study populations. Although between-study heterogeneity and methodological limitations resulted in very low certainty of the evidence, the synthesis consolidates previously dispersed findings and provides a reference for understanding the frequency of both phenotypes across different population and measurement contexts.

The treatment-denominator analysis further clarifies the interpretation of INH prevalence. JingNing and KoGES identified INH among untreated participants but used analytical denominators that also included treated participants, whereas the Jackson Heart Study and Rhee used untreated samples. Using reported non-treated denominators increased the pooled INH proportion from 17.3% to 19.6%, demonstrating how the choice of denominator affects the summarized frequency. Presenting these analyses together helps readers distinguish estimates based on mixed-treatment samples from those based on reported non-treated denominators. Differences in voluntary ABPM participation, age eligibility, and complete-case or imaging requirements also characterize the populations underlying each estimate. This study-level information complements the pooled results and facilitates comparisons between samples with similar recruitment and treatment criteria (8–10, 13).

### Relation to clinical evidence

The observed frequencies are consistent with the primary studies that established INH as a distinct ambulatory phenotype, while their variation is also clinically plausible (8–10). NH concerns absolute nighttime pressure; non-dipping and reverse/riser concern the night-to-day pattern. Neither can substitute for the other. European and Chinese consensus documents support ABPM-based nighttime assessment, but an elevated prevalence alone does not establish the benefit of a new screening strategy (4, 5).

Clinical and occupational reports provide context rather than a single reference population. Referral indication, age, antihypertensive treatment, diabetes, chronic kidney disease, obesity, and obstructive sleep apnea can differ substantially between such samples. Intermittent hypoxia, sleep fragmentation, and sympathetic activation make obstructive sleep apnea a plausible contributor to elevated nighttime pressure (21). These explanations were not quantified as independent effects in this review; they support descriptive stratification rather than an uninterpretable pooled clinical prevalence.

### Uncertainty and methodological implications

REML with modified Hartung–Knapp inference addresses uncertainty when few heterogeneous estimates are available, but prediction intervals remain approximate (16). The two-stage source analysis also depends on the estimated first-stage variance. Similar point estimates across transformations or leave-one-out analyses do not resolve between-population heterogeneity.

## Limitations

Selection and applicability limit interpretation: the source studies represent restricted communities or ABPM substudies, and treatment eligibility and INH classification differ. Adult-specific counts were unavailable for JingNing. The six IDACO event counts were retained from the previous extraction and could not be independently checked against the original nighttime data (Supplementary Table S7).

ABPM protocols varied in their definitions of night, sampling intervals, and recording-quality requirements. The cumulative impact of post-protocol modifications remains an important limitation, particularly the relaxation of ABPM eligibility to accept lower study-specific nighttime minima. No extractable numerator and denominator demonstrated both original requirements (≥70% successful nighttime readings and ≥10 valid nighttime measurements), so the strict-protocol sensitivity analysis could not be performed. The influence of this relaxation on the pooled proportions therefore remains unresolved. Confidence and prediction intervals reflect uncertainty under the selected model rather than correction of selection or measurement bias.

The broadened clinical and circadian evidence was not supplemented by a new search or re-screening, so it remains non-exhaustive. The effect of relaxed ABPM eligibility on earlier exclusions could not be determined. Protocol amendments and their timing were incompletely documented, and gray literature was not searched.

### Clinical and research implications

This review brings together the available evidence on NH and INH, providing a quantitative synthesis of their frequency in population-based and community samples. By presenting pooled estimates alongside individual-study findings, it offers clinicians and researchers a consolidated reference that would otherwise require examining multiple separate publications. The synthesis also makes differences in study populations, antihypertensive treatment status, and ABPM protocols explicit, helping readers interpret the reported prevalences and compare findings from similar populations. These summarized data provide a basis for understanding the frequency of nocturnal blood pressure abnormalities and evaluating targeted detection strategies using ABPM. Clear reporting of treatment-specific denominators, distinctions between NH and dipping patterns, nighttime recording completeness, and participation would further improve comparability across studies.

## Conclusions

NH was common in the population-based and community samples studied, with a pooled prevalence of 42.5%, while the pooled proportion of INH was 17.3%, according to the definitions used in the included studies. These findings highlight the importance of elevated nighttime blood pressure, including its occurrence in individuals with normal daytime blood pressure. Interpretation of the results should take into account the very low certainty of the evidence, as well as the relaxation of the quality criteria for ABPM, the effect of which could not be evaluated in the strict-protocol sensitivity analysis. This synthesis provides reference estimates to quantify the frequency of both phenotypes, inform the planning of population-based studies, and guide the evaluation of targeted detection strategies using ABPM.

## Data Availability

The original contributions presented in the study are included in the article/Supplementary Material; further inquiries can be directed to the corresponding author.
