# Supplementary material for: Prevalence of nocturnal and isolated nocturnal hypertension in population-based studies: a systematic review and meta-analysis
Source: Front Cardiovasc Med. 2026 Oct 28;13:1883404. doi: 10.3389/fcvm.2026.1883404 (PMC13751598; doi:10.3389/fcvm.2026.1883404)
Supplement: Supplementary file 1 [file Datasheet1.docx]

Supplementary Material

Prevalence of Nocturnal and Isolated Nocturnal Hypertension in Population-Based Studies: A Systematic Review and Meta-Analysis

Table S1. PRISMA 2020 checklist.

Table S2. Protocol amendments and methodological decisions.

Table S3a. Search strategies.

Table S3b. Disposition of full-text reports.

Tables S4a–S4b. Individual population NH and study-defined INH estimates.

Tables S4c–S4h. Individual contextual reports; no pooled estimates.

Table S5. Population model comparisons and source-level sensitivity.

Tables S6a–S6b. Nighttime recording quality and INH treatment denominators.

Tables S7a–S7b. JBI 2017 assessment and item-level justifications.

Table S8. GRADE-informed certainty judgments.

Tables S9a–S9b. Source mapping and contextual-report interpretation.

Supplementary references.

Figures S1–S2. Source-level NH and treatment-denominator INH sensitivities.

Figures S3–S6. Clinical/occupational and circadian observations without pooled estimators.

Table S1. PRISMA 2020 checklist.

| **Section / topic** | **Item** | **PRISMA 2020 reporting item** | **Location in the manuscript** |
| --- | --- | --- | --- |
| Title | 1 | Identify the report as a systematic review. | Manuscript title. |
| Abstract | 2 | See the PRISMA 2020 checklist for abstracts (PRISMA-A). | Abstract. |
| Rationale | 3 | Describe the rationale for the review in the context of existing knowledge. | Introduction. |
| Objectives | 4 | Provide an explicit statement of the objective(s) or question(s) addressed by the review. | Introduction, final paragraph. |
| Eligibility criteria | 5 | Specify the inclusion and exclusion criteria for the review and how studies were grouped for the syntheses. | Methods, Eligibility and outcome definitions; Table S2. |
| Information sources | 6 | Specify all databases, registers, websites, organizations, reference lists, and other sources searched or consulted to identify studies. Specify the date when each source was last searched. | Methods, Information sources and search strategy. |
| Search strategy | 7 | Present the full search strategies for all databases, registers, and websites, including any filters and limits used. | Table S3a. |
| Selection process | 8 | Specify the methods used to decide whether a study met the review inclusion criteria, including how many reviewers screened each record and each retrieved report, whether they worked independently, and details of automation tools used in the process, if applicable. | Methods, Study selection and data extraction; Protocol registration and scope. |
| Data extraction process | 9 | Specify the methods used to extract data from reports, including how many reviewers extracted data from each report, whether they worked independently, processes for obtaining or confirming data from study investigators, and details of automation tools used, if applicable. | Methods, Study selection and data extraction. |
| Data: outcomes | 10a | List and define all outcomes for which data were sought. Specify whether all results compatible with each outcome domain were sought in each study, and if not, how the results to be reported were selected. | Methods, Eligibility and outcome definitions; Treatment denominators for INH. |
| Data: other variables | 10b | List and define all other variables for which data were sought (e.g., participant characteristics, interventions, funding sources). Describe any assumptions made about missing or unclear information. | Methods, Study selection and data extraction; Tables 1 and S6. |
| Risk of bias in studies | 11 | Specify the methods used to assess risk of bias in included studies, including tools used, how many reviewers assessed each study and whether they worked independently, and details of automation tools used, if applicable. | Methods, Risk-of-bias assessment. |
| Effect measures | 12 | Specify for each outcome the effect measures used in the synthesis or presentation of results. | Methods, Statistical synthesis and uncertainty. |
| Synthesis methods | 13a | Describe the processes used to decide which studies were eligible for each synthesis. | Methods, Protocol registration and scope; Table S3b. |
| Synthesis methods | 13b | Describe methods required to prepare data for presentation or synthesis, such as handling missing data or data transformations. | Methods, Study selection and data extraction; Treatment denominators for INH. |
| Synthesis methods | 13c | Describe methods used to tabulate or visually display results of individual studies and syntheses. | Tables 1–5 and S4–S9; Figures 1–4 and S1–S6. |
| Synthesis methods | 13d | Describe methods used to synthesize results and provide a rationale for the choice. If meta-analysis was performed, describe the model(s), methods used to identify the presence and extent of statistical heterogeneity, and software packages used. | Methods, Statistical synthesis and uncertainty; Table S5. |
| Synthesis methods | 13e | Describe any methods used to explore possible causes of heterogeneity among study results. | Methods, Statistical synthesis and uncertainty. Meta-regressions were withdrawn. |
| Synthesis methods | 13f | Describe any sensitivity analyses conducted to assess the robustness of synthesized results. | Methods, Statistical synthesis and uncertainty; Tables 5, S5, S6 and S9a. |
| Assessment of reporting bias | 14 | Describe methods used to assess risk of bias due to missing results in a synthesis arising from reporting bias. | Methods, Reporting bias and certainty. |
| Certainty assessment | 15 | Describe methods used to assess certainty (or confidence) in the body of evidence for an outcome. | Methods, Reporting bias and certainty; Table S8. |
| Study selection | 16a | Describe the results of the search and selection process, from the number of records identified to the number of studies included, ideally using a flow diagram. | Results, Selection and revised analytical disposition; Figure 1. |
| Excluded studies | 16b | Cite studies that might appear to meet inclusion criteria but were excluded, and explain why. | Table S3b. |
| Study characteristics | 17 | Cite each included study and present its characteristics. | Table 1; Tables S3b and S9b. |
| Risk of bias in studies | 18 | Present risk-of-bias assessments for each included study. | Table 2; Figure 2; Tables S7a–S7b. |
| Results of individual studies | 19 | For all outcomes, present for each study: (a) point estimate and 95% CI and (b) structured graphical presentation, ideally a forest plot. | Tables S4a–S4h; Figures 3–4 and S1–S6. |
| Results of syntheses | 20a | For each synthesis, briefly summarize the characteristics and risk of bias of contributing studies. | Results, Population sources and comparability; Tables 1–3. |
| Results of syntheses | 20b | Present results of all statistical syntheses. If meta-analysis was performed, present for each the summary estimate and precision and measures of statistical heterogeneity. | Results, Population quantitative results; Tables 4 and S5; Figures 3–4. |
| Results of syntheses | 20c | Present results of all investigations of possible causes of heterogeneity among study results. | Meta-regressions were withdrawn; individual study characteristics are reported in Tables 1 and S4. |
| Results of syntheses | 20d | Present results of all sensitivity analyses conducted to assess robustness of synthesized results. | Results, Sensitivity and recording-quality audit; Tables 5, S5–S6 and S9a. |
| Reporting biases | 21 | Present assessments of risk of bias due to missing results arising from reporting bias for each synthesis assessed. | Methods, Reporting bias and certainty; Table S8. |
| Certainty of evidence | 22 | Present certainty assessments for each outcome evaluated. | Table 3; Table S8. |
| Discussion | 23a | Provide a general interpretation of the results in the context of other evidence. | Discussion, Principal findings and interpretation; Relation to clinical evidence. |
| Discussion | 23b | Discuss limitations of the evidence included in the review. | Discussion, Limitations. |
| Discussion | 23c | Discuss limitations of the review processes used. | Discussion, Limitations; Table S2. |
| Discussion | 23d | Discuss implications of the results for practice, policy, and future research. | Discussion, Clinical and research implications; Conclusions. |
| Registration and protocol | 24a | Provide registration information for the review, including registry name and registration number, or state that the review was not registered. | Methods and Declarations: PROSPERO CRD420251233166. |
| Registration and protocol | 24b | Indicate where the review protocol can be accessed, or state that no protocol was prepared. | Declarations, Protocol registration: PROSPERO CRD420251233166. |
| Registration and protocol | 24c | Describe and explain any amendments to information provided at registration or in the protocol. | Methods; Table S2. |
| Support | 25 | Describe sources of financial or non-financial support for the review and the role of funders or sponsors. | Declarations, Funding. |
| Conflicts of interest | 26 | Declare any conflicts of interest of the review authors. | Declarations, Conflicts of interest. |
| Availability of data, code, and other materials | 27 | Report which of the following are publicly available and where they can be found: data-collection form templates; data extracted from included studies; data used for all analyses; analytic code; and any other materials used in the review. | Declarations, Data availability. Aggregate data are provided in the Supplementary Material. |

Table S2. Protocol amendments and methodological decisions.

| **Domain** | **Protocol plan** | **Previously submitted implementation** | **Current implementation** | **Timing and implications** |
| --- | --- | --- | --- | --- |
| Population and selection | General/community adults; nonrepresentative occupational samples excluded. | Clinical, occupational and high-risk reports added during full-text review. | Five population/community sources form the quantitative core; twelve other previously retained reports are contextual/non-extractable. | Eligibility expanded during full-text review; re-screening under the broadened criteria was not documented. |
| Secondary outcomes and search | INH and dipping listed as additional outcomes when reported. | INH and circadian results pooled across separately labeled strata. | INH remains a population synthesis. Circadian reports are presented individually as contextual evidence. | INH and dipping were prespecified; outcome-specific search coverage remains limited. |
| Nighttime recording quality | ≥70% successful NIGHTTIME readings and ≥10 valid NIGHTTIME measurements. | Lower study-specific nighttime minima accepted. | Source-specific audit distinguishes nighttime from 24-hour validity. A strict-protocol pooled sensitivity is not estimable. | Timing relative to interim results was not documented. No extractable denominator demonstrated both original criteria. The effect of relaxing these requirements on the pooled proportions remains unresolved and is considered with the other post-protocol modifications. |
| Age restriction | Adults aged ≥18 years. | JingNing’s published sample retained eight adolescents. | The deviation is explicit; whole-study omission is reported for both outcomes. No adult-only events are imputed. | Identified in source audit. Adult-only counts are not recoverable from the available report. |
| Statistical model | Freeman–Tukey with DerSimonian–Laird; alternative transformations and τ² estimators as sensitivities. | Logit with DL and normal confidence intervals promoted to primary presentation. | Logit–REML with modified Hartung–Knapp is the current primary model; DL-normal and Freeman–Tukey are comparisons. | REML/mKH promotion responds to this editorial round. Earlier hierarchy-change timing relative to interim results cannot be established. |
| Appraisal instrument | Ten-criterion Munn-based implementation with numerical categories. | Adapted D1–D9 framework; applicability separated. | Nine-item JBI 2017 with original response categories and source-linked rationales; no summed score. Three authors assessed the sources, with fourth-author adjudication; AI-assisted organization and content checks were verified by the authors, who made all final judgments. | Instrument and implementation revised during the second editorial round. |
| Core-domain rule | No D5–D8 exclusion rule in the local protocol. | Post hoc D5–D8 exclusion sensitivity introduced. | Rule and associated pooled results withdrawn. | Post hoc rule introduced in the previous revision and now withdrawn. |
| IDACO structure | Avoid participant duplication. | Six IDACO rows plus three other NH sources. | 6,357/6,546 IDACO participants after removing 189 from JingNing; EPOGH identified as four cohorts within one analytical component. | The total NH denominator remains 8,282. Two-stage source-level and IDACO-omission sensitivities assess influence. |
| INH denominators | Population proportions; treatment stratification if data permit. | Published analytical denominators combined despite different treatment rules. | Original-denominator pooled proportion is explicitly descriptive. A separate non-current-treatment denominator sensitivity uses N=3,061. | Second-round audit. Does not harmonize current versus historical treatment, adolescent eligibility, or selection. |
| Meta-regression | Publication-year exploration and subgroup analyses conditional on data. | Regional, setting and other univariable models reported. | All meta-regressions withdrawn; geography and sampling remain descriptive. | Insufficient replicated and balanced contrasts; no adjusted regional or causal-setting inference remains. |
| Certainty | GRADE adapted to prevalence; no present numerical CI-width rules. | Revision-generated thresholds and core-domain judgments used. | Outcome-level GRADE-informed assessment without summed JBI or automatic ten-point CI-width thresholds. Both summaries: very low certainty. | Revision-stage judgments, not original protocol specifications. |
| Reporting-bias tests | Funnel, Egger and trim-and-fill planned, with acknowledged limitations. | Earlier broad cross-setting tests subsequently withdrawn. | No formal asymmetry tests retained; no pooling across settings to reach a numerical k threshold. | Too few independent sources in each coherent synthesis. |

Table S3a. Search strategies.

Last search date: March 17, 2026.

DATABASE 1: PubMed/MEDLINE

((("Hypertension"[Mesh]) AND "Circadian Rhythm"[Mesh]) OR "nocturnal hypertension"[tiab] OR "night-time hypertension"[tiab] OR "nighttime hypertension"[tiab] OR "sleep hypertension"[tiab] OR "night blood pressure"[tiab] OR "nighttime blood pressure"[tiab] OR "nocturnal blood pressure"[tiab] OR "sleep blood pressure"[tiab] OR "nocturnal BP"[tiab] OR "night-time BP"[tiab] OR "nighttime BP"[tiab])

AND

(("Blood Pressure Monitoring, Ambulatory"[Mesh]) OR "ambulatory blood pressure"[tiab] OR "ABPM"[tiab] OR "24-hour blood pressure"[tiab] OR "24-h blood pressure"[tiab] OR "24 hour blood pressure"[tiab] OR "blood pressure monitoring"[tiab])

AND

(("Prevalence"[Mesh]) OR "prevalence"[tiab] OR "frequency"[tiab] OR "epidemiology"[tiab] OR "distribution"[tiab])

DATABASE 2: Embase

'hypertension'/exp OR 'nocturnal hypertension':ab,ti OR 'night-time hypertension':ab,ti OR 'nighttime hypertension':ab,ti OR 'night time hypertension':ab,ti OR 'sleep hypertension':ab,ti OR 'night blood pressure':ab,ti OR 'nighttime blood pressure':ab,ti OR 'night-time blood pressure':ab,ti OR 'night time blood pressure':ab,ti OR 'nocturnal blood pressure':ab,ti OR 'sleep blood pressure':ab,ti OR 'nocturnal bp':ab,ti OR 'night-time bp':ab,ti OR 'nighttime bp':ab,ti OR 'night time bp':ab,ti OR 'non-dipper':ab,ti OR 'nondipping':ab,ti OR 'non dipping':ab,ti OR 'reverse dipper':ab,ti OR 'extreme dipper':ab,ti OR 'dipping status':ab,ti

AND

'ambulatory blood pressure monitoring'/exp OR 'ambulatory blood pressure':ab,ti OR 'abpm':ab,ti OR '24-hour blood pressure':ab,ti OR '24-h blood pressure':ab,ti OR '24 hour blood pressure':ab,ti OR 'blood pressure monitoring':ab,ti

AND

'prevalence'/exp OR 'prevalence':ab,ti OR 'frequency':ab,ti OR 'epidemiology':ab,ti OR 'incidence':ab,ti

DATABASE 3: Scopus

TITLE-ABS-KEY("nocturnal hypertension" OR "night-time hypertension" OR "nighttime hypertension" OR "sleep hypertension" OR "night blood pressure" OR "nighttime blood pressure" OR "nocturnal blood pressure" OR "sleep blood pressure" OR "nocturnal BP" OR "night-time BP" OR "nighttime BP")

AND

TITLE-ABS-KEY("ambulatory blood pressure" OR "ABPM" OR "24-hour blood pressure" OR "24-h blood pressure" OR "24 hour blood pressure" OR "blood pressure monitoring")

AND

TITLE-ABS-KEY("prevalence" OR "frequency" OR "epidemiology" OR "distribution")

DATABASE 4: Web of Science Core Collection

TS=("nocturnal hypertension" OR "night-time hypertension" OR "nighttime hypertension" OR "sleep hypertension" OR "night blood pressure" OR "nighttime blood pressure" OR "nocturnal blood pressure" OR "sleep blood pressure" OR "nocturnal BP" OR "night-time BP" OR "nighttime BP")

AND

TS=("ambulatory blood pressure" OR "ABPM" OR "24-hour blood pressure" OR "24-h blood pressure" OR "24 hour blood pressure" OR "blood pressure monitoring")

AND

TS=("prevalence" OR "frequency" OR "epidemiology" OR "distribution")

Table S3b. Disposition of full-text reports.

| **Report** | **Original report type** | **Current use** | **Reason / denominator restriction** |
| --- | --- | --- | --- |
| Melgarejo/IDACO 2017 [1] | Original collaborative population study | Population quantitative source | NH; six analytical components. IDACO JingNing was excluded to avoid overlap. |
| Li/JingNing 2007 [2] | Original rural population study | Population quantitative source | NH and INH; published denominator retains eight adolescents and selected treated participants. |
| Ogedegbe/JHS 2013 [3] | Original African-American population study | Population quantitative source | NH and INH in untreated ABPM volunteers; > rather than ≥ threshold boundary. |
| Rhee/Korea 2021 [4] | Original population study | Population quantitative source | NH and INH among untreated adults; mixed recruitment mechanism. |
| Kim/KoGES 2022 [5] | Original community/KoGES sub-sample | Population quantitative source | Study-defined INH in an ABPM/MRI subgroup; mixed treatment denominator. |
| Omboni/TEMPLAR 2019 [6] | Community-pharmacy registry | Contextual / not pooled | Pharmacy-referred ABPM; INH count is approximate. |
| Etyang/Kenya 2016 [7] | Original population diagnostic study | Contextual / not pooled | Population ABPM, but no separable nighttime numerator for the prioritized outcome. |
| Melgarejo/Maracaibo 2022 [8] | Original population/MRI sub-sample | Contextual / not pooled | Population-derived MRI subset using ≥110/65; not combined with 120/70. |
| Amjo/Nigeria 2020 [9] | Original clinical study in hypertensive patients | Contextual / not pooled | Referred hypertensive clinical sample, not a population prevalence denominator. |
| de la Sierra/Spain ABPM 2014 [10] | Clinical registry | Contextual / not pooled | Clinical registry; systolic-only ≥120 and dipping. Treatment strata displayed separately. |
| Barochiner/Buenos Aires 2023 [11] | Original tertiary hospital study | Contextual / not pooled | Tertiary-hospital clinical sampling; individual proportions only. |
| Banegas/MUCH 2014 [12] | Treated clinical registry | Contextual / not pooled | Masked uncontrolled hypertension conditional on treated/clinic-controlled status. |
| Minetto/La Plata 2026 [13] | Original outpatient clinical study | Contextual / not pooled | Cardiometabolic outpatients; referral-based population. |
| Chuwa/Tanzania 2020 [14] | Original occupational study | Contextual / not pooled | University employees; occupational recruitment. |
| Huang/Shanghai 2022 [15] | Nested case-control/source clinical population | Contextual / not pooled | Clinic source population 155/1,368; no use of case-control denominator. |
| Kaul/India ABPM 2019 [16] | Clinical all-comers registry | Contextual / not pooled | Clinically referred registry; circadian phenotypes only. |
| Kario/JAMP 2020 [17] | High-risk clinical cohort | Contextual / not pooled | High-risk clinical cohort; individual NH/circadian proportions only. |
| Staplin/Lancet 2023 [18] | Clinical prognostic cohort | Historical exclusion / background only | Prognostic cohort; no extractable prioritized NH prevalence. |
| Doyle/North Texas 2019 [19] | Original objective sleep/continuous BP study | Historical exclusion / background only | Continuous nighttime BP and sleep, without extractable NH phenotype counts. |
| Parati/ESH 2025 [20] | Position paper | Historical exclusion / background only | Position paper without original prevalence data. |
| Liu/Chinese consensus 2024 [21] | Consensus/guideline | Historical exclusion / background only | Consensus document without original prevalence data. |
| Cuspidi/reverse dipping 2017 [22] | Review | Historical exclusion / background only | Narrative review; not an original quantitative source. |
| Kim/KoGES TOD 2016 [23] | Original study without nocturnal ABPM | Historical exclusion / background only | No ABPM/nighttime measurement for the prioritized outcome. |

Table S4a. Individual NH estimates in population/community samples.

| **Estimate / source** | **Country** | **Sampling setting** | **Threshold** | **Cases / N** | **Prevalence %, Wilson 95% CI** |
| --- | --- | --- | --- | --- | --- |
| IDACO Copenhagen 2017 [1] | Denmark | Population cohort | ≥120/70 | 859/2,142 | 40.1 (38.0–42.2) |
| IDACO Ohasama 2017 [1] | Japan | Population cohort | ≥120/70 | 368/1,275 | 28.9 (26.4–31.4) |
| IDACO Noorderkempen 2017 [1] | Belgium | Population cohort | ≥120/70 | 233/943 | 24.7 (22.1–27.6) |
| IDACO Montevideo 2017 [1] | Uruguay | Population cohort | ≥120/70 | 415/962 | 43.1 (40.0–46.3) |
| IDACO EPOGH 2017 [1] | Europe mixed | Population cohort | ≥120/70 | 191/491 | 38.9 (34.7–43.3) |
| IDACO Maracaibo 2017 [1] | Venezuela | Population cohort | ≥120/70 | 346/544 | 63.6 (59.5–67.5) |
| Li 2007 JingNing total NH [2] | China | Population rural | ≥120/70 | 334/677 | 49.3 (45.6–53.1) |
| Ogedegbe 2013 JHS total NH [3] | USA | Population cohort; African-American untreated | >120 or >70 | 233/425 | 54.8 (50.1–59.5) |
| Rhee 2021 Korea total NH untreated [4] | South Korea | General population; untreated | ≥120/70 | 352/823 | 42.8 (39.4–46.2) |

IDACO contributes 6,357 participants after exclusion of its 189 JingNing participants. EPOGH combines four cohorts. The six IDACO event counts were retained from the previous extraction and could not be independently checked against original Table S7. JingNing includes eight adolescents and selected treated participants; JHS uses >120 or >70 mmHg rather than ≥.

Table S4b. Study-defined INH proportions using original analytical denominators.

| **Estimate / source** | **Country** | **Sampling setting** | **Threshold** | **Cases / N** | **Prevalence %, Wilson 95% CI** |
| --- | --- | --- | --- | --- | --- |
| Li 2007 JingNing INH [2] | China | Population rural | ≥120/70 | 74/677 | 10.9 (8.8–13.5) |
| Ogedegbe 2013 JHS INH [3] | USA | Population cohort; African-American untreated | >120 or >70 | 81/425 | 19.1 (15.6–23.1) |
| Rhee 2021 Korea INH [4] | South Korea | General population; untreated | ≥120/70 | 188/823 | 22.8 (20.1–25.8) |
| Kim 2022 KoGES INH [5] | South Korea | KoGES community; MRI/ABPM subset | ≥120/70 | 314/1,734 | 18.1 (16.4–20.0) |

These denominators are not uniformly untreated. JingNing includes 71 treated participants with day–night hypertension; KoGES includes 527 medication users although the INH definition excludes treatment history. JHS and Rhee exclude current medication. See Table S6b for the separate denominator sensitivity.

Table S4c. Individual contextual NH observations in clinical, occupational and high-risk samples.

| **Estimate / source** | **Country** | **Sampling setting** | **Threshold** | **Cases / N** | **Prevalence %, Wilson 95% CI** |
| --- | --- | --- | --- | --- | --- |
| Amjo 2020 Nigeria NH [9] | Nigeria | Clinical hypertensive referred | ≥120/70 | 61/77 | 79.2 (68.9–86.8) |
| Barochiner 2023 BuenosAires total NH [11] | Argentina | Tertiary hospital hypertensive ABPM | ≥120/70 | 319/917 | 34.8 (31.8–37.9) |
| Minetto 2026 LaPlata total NH [13] | Argentina | Cardiometabolic outpatient | ≥120/70 | 1,130/2,297 | 49.2 (47.2–51.2) |
| Chuwa 2020 Tanzania NH [14] | Tanzania | Occupational university employees | ≥120/70 | 214/390 | 54.9 (49.9–59.7) |
| Kario 2020 JAMP NH [17] | Japan | Clinical high-risk/general practice | ≥120/70 | 3,968/6,359 | 62.4 (61.2–63.6) |

Contextual reports; no pooled estimate.

Table S4d. Individual contextual INH observations.

| **Estimate / source** | **Country** | **Sampling setting** | **Threshold** | **Cases / N** | **Prevalence %, Wilson 95% CI** |
| --- | --- | --- | --- | --- | --- |
| Barochiner 2023 BuenosAires INH [11] | Argentina | Tertiary hospital hypertensive ABPM | ≥120/70 | 144/917 | 15.7 (13.5–18.2) |
| Minetto 2026 LaPlata INH [13] | Argentina | Cardiometabolic outpatient | ≥120/70 | 383/2,297 | 16.7 (15.2–18.3) |
| Huang 2022 China INH source [15] | China | Hypertension clinic; source population | ≥120/70 | 155/1,368 | 11.3 (9.8–13.1) |
| Chuwa 2020 Tanzania INH [14] | Tanzania | Occupational university employees | ≥120/70 | 157/390 | 40.3 (35.5–45.2) |
| TEMPLAR 2019 Italy INH approx [6] | Italy | Community pharmacy ABPM referral | ≥120/70 | ≈3,261/20,773 | ≈15.7 (15.2–16.2) |

TEMPLAR’s numerator is approximate; the displayed Wilson interval is conditional on that reconstructed count and does not incorporate reconstruction uncertainty. Huang uses the clinic source denominator, not the case-control subset.

Table S4e. Contextual NH under the alternative ≥110/65 mmHg threshold.

| **Estimate / source** | **Country** | **Sampling setting** | **Threshold** | **Cases / N** | **Prevalence %, Wilson 95% CI** |
| --- | --- | --- | --- | --- | --- |
| Melgarejo 2022 Maracaibo NH ACC AHA [8] | Venezuela | Population-based MRI/ABPM subset | ≥110/65 | 292/429 | 68.1 (63.5–72.3) |

The ≥110/65 mmHg definition was not combined with the primary 120/70 mmHg threshold.

Table S4f. Contextual systolic-only NH, separated by treatment status.

| **Estimate / source** | **Country** | **Sampling setting** | **Threshold** | **Cases / N** | **Prevalence %, Wilson 95% CI** |
| --- | --- | --- | --- | --- | --- |
| DeLaSierra 2014 Spain NH SBP untreated [10] | Spain | Clinical hypertensive registry untreated | Night SBP ≥120 | 15,187/37,096 | 40.9 (40.4–41.4) |
| DeLaSierra 2014 Spain NH SBP treated [10] | Spain | Clinical hypertensive registry treated | Night SBP ≥120 | 31,277/62,788 | 49.8 (49.4–50.2) |

The two treatment strata belong to the same Spanish registry. The definition uses nighttime systolic pressure only.

Table S4g. Contextual non-dipping observations, without pooling.

| **Estimate / source** | **Country** | **Sampling setting** | **Threshold** | **Cases / N** | **Prevalence %, Wilson 95% CI** |
| --- | --- | --- | --- | --- | --- |
| Amjo 2020 Nigeria Nondip [9] | Nigeria | Clinical hypertensive referred | Not harmonized | 43/77 | 55.8 (44.7–66.4) |
| Chuwa 2020 Tanzania Nondipping [14] | Tanzania | Occupational | Not harmonized | 260/390 | 66.7 (61.8–71.2) |
| Kaul 2019 India Nondipper [16] | India | Clinical all-comers ABPM | Not harmonized | 12,532/27,472 | 45.6 (45.0–46.2) |
| Kario 2020 JAMP Nondipper [17] | Japan | Clinical high-risk | Not harmonized | 2,077/6,359 | 32.7 (31.5–33.8) |
| DeLaSierra 2014 Spain Nondip untreated [10] | Spain | Clinical hypertensive registry untreated | Not harmonized | 16,506/37,096 | 44.5 (44.0–45.0) |
| DeLaSierra 2014 Spain Nondip treated [10] | Spain | Clinical hypertensive registry treated | Not harmonized | 35,925/62,788 | 57.2 (56.8–57.6) |

Definitions and sampling frames differ between reports. Non-dipping was not pooled or treated as equivalent to absolute-threshold NH.

Table S4h. Contextual reverse/riser observations, without pooling.

| **Estimate / source** | **Country** | **Sampling setting** | **Threshold** | **Cases / N** | **Prevalence %, Wilson 95% CI** |
| --- | --- | --- | --- | --- | --- |
| Amjo 2020 Nigeria Reverse [9] | Nigeria | Clinical hypertensive referred | Not harmonized | 12/77 | 15.6 (9.1–25.3) |
| Chuwa 2020 Tanzania Reverse [14] | Tanzania | Occupational | Not harmonized | 94/390 | 24.1 (20.1–28.6) |
| Kaul 2019 India Reverse [16] | India | Clinical all-comers ABPM | Not harmonized | 3,549/27,472 | 12.9 (12.5–13.3) |
| Kario 2020 JAMP Riser [17] | Japan | Clinical high-risk | Not harmonized | 758/6,359 | 11.9 (11.1–12.7) |

Contextual observations without a pooled estimate.

Table S5. Population model comparisons and source-level sensitivity.

| **Outcome / dataset** | **Specification** | **k / sources** | **N** | **Pooled %, 95% CI** | **I², %** | **τ²** | **Approximate 95% PI, %** |
| --- | --- | --- | --- | --- | --- | --- | --- |
| NH; original counts | Logit–DL; normal CI | 9 / 4 | 8,282 | 42.5 (35.4–49.9) | 97.6 | 0.2023 | 19.4–69.4 |
| NH; original counts | Logit–REML; normal CI | 9 / 4 | 8,282 | 42.5 (34.6–50.8) | 97.6 | 0.2569 | 17.3–72.3 |
| NH; original counts | Logit–REML; modified Hartung–Knapp | 9 / 4 | 8,282 | 42.5 (33.2–52.3) | 97.6 | 0.2569 | 17.2–72.3 |
| NH; original counts | Freeman–Tukey–DL; normal CI | 9 / 4 | 8,282 | 42.6 (35.4–50.0) | 97.8 | 0.0125 | Not back-transformed |
| Study-defined INH; original counts | Logit–DL; normal CI | 4 / 4 | 3,659 | 17.4 (13.3–22.3) | 91.5 | 0.0914 | 4.6–47.8 |
| Study-defined INH; original counts | Logit–REML; normal CI | 4 / 4 | 3,659 | 17.3 (12.8–23.1) | 91.5 | 0.1257 | 3.6–53.9 |
| Study-defined INH; original counts | Logit–REML; modified Hartung–Knapp | 4 / 4 | 3,659 | 17.3 (10.4–27.5) | 91.5 | 0.1257 | 3.6–54.0 |
| Study-defined INH; original counts | Freeman–Tukey–DL; normal CI | 4 / 4 | 3,659 | 17.5 (13.0–22.5) | 92.4 | 0.0037 | Not back-transformed |
| NH; two-stage source weighting | Logit–DL; normal CI | 4 / 4 | 8,282 | 47.1 (41.1–53.3) | 85.1 | 0.0504 | 22.8–72.9 |
| NH; two-stage source weighting | Logit–REML; modified Hartung–Knapp | 4 / 4 | 8,282 | 47.3 (37.1–57.8) | 84.5 | 0.0533 | 22.2–73.8 |

REML = restricted maximum likelihood; DL = DerSimonian–Laird. Modified Hartung–Knapp uses q*=max(1,q), t(k−1) for the pooled CI, and the adjusted pooled variance. Approximate prediction intervals use t(k−2). I² is Q-based. Freeman–Tukey τ² is on the half-double-arcsine scale and cannot be compared directly with logit τ²; its prevalence-scale prediction interval is not reported. Its inverse uses the harmonic-mean sample size. These comparisons alter analysis, not the original eligibility or treatment history [24,25,26].

A separate INH denominator sensitivity includes 657 cases among 3,061 participants: 19.6 (11.4–31.6)%; approximate prediction interval 3.6–61.5%. The two-stage source model conditions on an estimated first-stage IDACO variance and is not a fully identified hierarchical model.

Table S6a. Nighttime recording quality relative to the original protocol.

| **Source** | **Nighttime minimum / schedule** | **Validity percentage** | **Both original nighttime criteria demonstrable?** |
| --- | --- | --- | --- |
| IDACO [1] | ≥5 nighttime; the ≥10 requirement in the source concerns daytime. | Nighttime percentage not separately reported. | Not guaranteed |
| Li [2] | ≥5 nighttime; programmed every 45 minutes at night. | Nighttime percentage not separately reported. | Not guaranteed |
| Ogedegbe [3] | ≥54 valid readings overall; no separate minimum nocturnal count. | ≥75% over 24 hours; nighttime percentage not specified. | Insufficient information |
| Rhee [4] | ≥7 nighttime; readings every 30 minutes. | >70% of total attempted measurements. | Not guaranteed |
| Kim [5] | ≥7 nighttime; hourly readings from 23:00 to 06:00. | >70% of total expected measurements. | Not guaranteed |

The protocol required ≥70% successful nighttime readings and ≥10 valid nighttime measurements. No extractable numerator and denominator documented both requirements, so the strict-protocol synthesis was not estimable. A lower study-specific minimum does not demonstrate that every recording failed the original criteria.

Table S6b. Treatment denominators for the INH sensitivity analysis.

| **Source** | **Original proportion** | **Denominator sensitivity** | **Reason and remaining distinction** |
| --- | --- | --- | --- |
| Li/JingNing [2] | 74/677 | 74/606 | Subtract 71 treated day–night cases; preserve the 74 untreated INH cases. Eight adolescents cannot be individually removed. |
| Jackson Heart Study [3] | 81/425 | 81/425 | The analytical sample already excludes current antihypertensive treatment. |
| Rhee/Korea [4] | 188/823 | 188/823 | The analytical sample already excludes current antihypertensive treatment. |
| Kim/KoGES [5] | 314/1,734 | 314/1,207 | Subtract 527 current medication users. The INH definition additionally requires no treatment history, which cannot be fully harmonized with current medication. |

The pooled proportion using original denominators was 17.3% (95% CI, 10.4–27.5); the post hoc sensitivity using reported non-current-treatment denominators was 19.6% (95% CI, 11.4–31.6). Treatment-history definitions were not fully harmonized.

Table S7a. JBI 2017 response matrix.

| **Source** | **Q1** | **Q2** | **Q3** | **Q4** | **Q5** | **Q6** | **Q7** | **Q8** | **Q9** |
| --- | --- | --- | --- | --- | --- | --- | --- | --- | --- |
| Melgarejo/IDACO 2017 [1] | Yes | Yes | Yes | Yes | Unclear | Yes | Unclear | Unclear | Unclear |
| Li/JingNing 2007 [2] | Yes | Unclear | Yes | Yes | No | Yes | Yes | No | Unclear |
| Ogedegbe/Jackson Heart Study 2013 [3] | Yes | No | Yes | Yes | No | Yes | Yes | No | No |
| Rhee/Korea 2021 [4] | Yes | No | Yes | Yes | Unclear | Yes | Yes | Yes | Unclear |
| Kim/KoGES 2022 [5] | Yes | Unclear | Yes | Yes | Unclear | Yes | Yes | No | Unclear |

Response options are Yes, No, Unclear, and Not applicable. Q1: sampling frame; Q2: sampling method; Q3: sample size; Q4: population and setting; Q5: analytical coverage; Q6: condition identification; Q7: measurement consistency; Q8: statistical analysis; Q9: response and non-response [27,28]. No summed score or D5–D8 exclusion rule was used. Applicability was assessed separately. Three authors assessed each source and verified the AI-assisted content against the available primary-study reports; a fourth author resolved discrepancies. AI assistance was limited to organizing checklist instructions and helping to check relevant study content. All final item-level judgments were made by the authors.

Table S7b. Item-level justifications: Melgarejo/IDACO 2017 [1].

| **Item** | **Judgment** | **Study-specific basis** |
| --- | --- | --- |
| Q1 — Sampling frame | Yes | Community cohorts with a defined analytical age range of 40–79 years. The sampling frame is judged against those communities, not the worldwide adult population. |
| Q2 — Sampling method | Yes | Participation in the collaboration required population cohorts with random recruitment. A collaborative publication is not treated as a single referral-based sample. |
| Q3 — Sample size | Yes | The source includes 6,546 participants and cohort sizes that permit informative overall proportions. This judgment does not establish precision for every subgroup or remove selection bias. |
| Q4 — Population and setting | Yes | Geographic origin, age, sex, treatment and cardiovascular risk factors are described. The sizes of the ten original cohorts are identified. |
| Q5 — Analytical coverage | Unclear | Exclusions for age, missing measurements and recording quality are described. Differential analytical coverage across groups within every cohort is not sufficiently documented. |
| Q6 — Condition identification | Yes | ABPM and diagnostic thresholds are explicit. The source’s definition incorporates antihypertensive treatment, whereas the extracted review outcome aims to count elevated nighttime pressure; these must be distinguished. |
| Q7 — Measurement consistency | Unclear | Devices and recording intervals differ across cohorts. The publication acknowledges methodological variation, so complete equivalence of measurement is not established. |
| Q8 — Statistical analysis | Unclear | The source describes its statistical methods. However, the exact derivation of the six elevated-nighttime-pressure counts requires original Supplementary Table S7, not present in the retrieved source file. This is outcome-extraction uncertainty, not proof of erroneous analysis in the original study. |
| Q9 — Response and non-response | Unclear | Initial participation rates and sufficiently detailed non-response analyses are not provided for every cohort in the retrieved report. Valid-record percentages do not replace recruitment-response information. |

Applicability, assessed separately: Restricted communities and ages 40–79 years; six extracted analytical components, including four EPOGH cohorts within one row. Not a worldwide probability sample.

Table S7b. Item-level justifications: Li/JingNing 2007 [2].

| **Item** | **Judgment** | **Study-specific basis** |
| --- | --- | --- |
| Q1 — Sampling frame | Yes | The source population comprises fourteen rural villages. Residents aged 12 years and older were invited; eight adolescents remained in the analytical sample, limiting applicability to an adult-only question. |
| Q2 — Sampling method | Unclear | Residents were invited, but the mechanism selecting the 733 participants undergoing ABPM from 1,490 consenting residents is not fully explained. |
| Q3 — Sample size | Yes | The analytical sample of 677 provides informative intervals for overall prevalence. It does not establish adequacy for every age or regional subgroup or a precision target prespecified by this review. |
| Q4 — Population and setting | Yes | The rural population, clinical characteristics, treatment rules, ABPM protocol and blood-pressure categories are described. |
| Q5 — Analytical coverage | No | Twenty-five treated participants with normal daytime or nighttime pressure were excluded, but 71 treated participants with day–night hypertension were retained. Analytical coverage therefore depends on phenotype and affects a population-prevalence interpretation. |
| Q6 — Condition identification | Yes | The ABPM device, thresholds and monthly calibration are identified. Total NH can be derived as 74 isolated cases plus 260 day–night cases, yielding 334. |
| Q7 — Measurement consistency | Yes | Recording intervals and fixed time windows are applied consistently. Minimum duration, validation of the nighttime interval and time-weighted averaging are described. |
| Q8 — Statistical analysis | No | No prevalence-specific confidence intervals accompany the NH/INH category proportions in the supplied article. The four category counts are reproducible (310 + 33 + 74 + 260 = 677), but recoverable arithmetic alone does not fully satisfy the JBI guidance for Q8. The review supplies Wilson intervals separately; this does not retroactively complete the source reporting. Phenotype-dependent selection is appraised in Q5. |
| Q9 — Response and non-response | Unclear | Initial participation was 1,490/2,059 (72.4%), but this is not the response rate for the ABPM substudy. Differential selection into ABPM cannot be excluded from the reported information. |

Applicability, assessed separately: Rural sample including eight adolescents and phenotype-dependent retention of treated participants; neither an unrestricted adult nor an exclusively untreated denominator.

Table S7b. Item-level justifications: Ogedegbe/Jackson Heart Study 2013 [3].

| **Item** | **Judgment** | **Study-specific basis** |
| --- | --- | --- |
| Q1 — Sampling frame | Yes | The source is a community cohort of noninstitutionalized African-American adults. The analytical population is further restricted to participants not taking antihypertensive medication. |
| Q2 — Sampling method | No | ABPM was offered to the cohort and accepted voluntarily by 1,150 of 5,301 participants; the ABPM substudy was not a probability sample of the parent cohort. |
| Q3 — Sample size | Yes | The sample of 425 gives informative intervals for overall proportions, although precision is lower than in larger cohorts and is not assured for every subgroup. |
| Q4 — Population and setting | Yes | Age, sex, ethnicity, treatment and eligibility are described, including differences between participants with and without ABPM. |
| Q5 — Analytical coverage | No | Volunteer participation and exclusions for invalid recordings or missing diaries reduced the analytical sample. Differences in age and sex between ABPM participants and nonparticipants were documented. |
| Q6 — Condition identification | Yes | ABPM used SpaceLabs 90207. The reported threshold was nighttime SBP >120 or DBP >70 mmHg, with normal daytime pressure for INH. The strict > rather than ≥ boundary is retained in the extraction. |
| Q7 — Measurement consistency | Yes | Readings were obtained every 20 minutes over 24 hours; trained personnel, sleep diaries and uniform recording-validity rules were used. |
| Q8 — Statistical analysis | No | The report supplies category counts (176 + 16 + 81 + 152 = 425) and percentages, but not NH/INH prevalence-specific confidence intervals. Confidence intervals for regression odds ratios concern different estimands. Q8 is therefore No for the source prevalence reporting, although the review can reconstruct valid Wilson intervals from the counts. |
| Q9 — Response and non-response | No | ABPM participation was low and participants differed in relevant characteristics. Comparison with nonparticipants describes, but does not demonstrate correction of, selection for prevalence estimation. |

Applicability, assessed separately: African-American adults without current antihypertensive treatment who volunteered for ABPM.

Table S7b. Item-level justifications: Rhee/Korea 2021 [4].

| **Item** | **Judgment** | **Study-specific basis** |
| --- | --- | --- |
| Q1 — Sampling frame | Yes | The initial sampling frame covered telephone numbers in five cities. The final analytical population was restricted to participants without current antihypertensive medication. |
| Q2 — Sampling method | No | Of 1,128 recruited participants, 971 followed invitations and 157 were relatives or friends of invited individuals. The final recruitment process was therefore not wholly probabilistic. |
| Q3 — Sample size | Yes | The 823 participants and explicit counts permit informative overall prevalence intervals. Adequacy is not assumed for all secondary comparisons. |
| Q4 — Population and setting | Yes | The cities, recruitment process, treatment status, participant characteristics, and office and ambulatory measurements are described. |
| Q5 — Analytical coverage | Unclear | Of 1,128 recruited participants, 823 were analyzed after treatment and ABPM-validity restrictions. The report does not fully establish differential coverage of all recruitment groups. |
| Q6 — Condition identification | Yes | ABPM used Mobil-O-Graph, with explicit nighttime ≥120/70 and daytime <135/85 mmHg thresholds for INH. |
| Q7 — Measurement consistency | Yes | Readings were obtained every 30 minutes using fixed time windows. Validity required >70% of total attempted measurements and at least 14 daytime and seven nighttime readings. |
| Q8 — Statistical analysis | Yes | NH (352 cases) and INH (188 cases) are reported within 823 untreated participants. The source also reports 95% margins of error of 3.4 percentage points for NH and 2.9 for INH, supplying uncertainty for these proportions. Categories and denominators are explicit. The etiological regression is not treated as the prevalence estimator. |
| Q9 — Response and non-response | Unclear | A complete denominator of people contacted and sufficient assessment of non-response to telephone recruitment and ABPM are not reported. |

Applicability, assessed separately: Urban participants without current treatment, recruited by a mixture of random telephone invitation and relatives/friends; not necessarily all Korean adults.

Table S7b. Item-level justifications: Kim/KoGES 2022 [5].

| **Item** | **Judgment** | **Study-specific basis** |
| --- | --- | --- |
| Q1 — Sampling frame | Yes | The source is a community cohort of middle-aged and older adults. The prevalence estimate comes from participants in an ABPM and brain-MRI substudy. |
| Q2 — Sampling method | Unclear | Probability sampling of the ABPM/MRI subgroup from the full community cohort is not demonstrated. |
| Q3 — Sample size | Yes | The sample of 1,734 with 314 INH cases supports an informative overall proportion. This does not remove selection restrictions or guarantee precision within each subgroup. |
| Q4 — Population and setting | Yes | Cohort visits, age, sex, clinical exclusions, treatment and the ABPM/imaging procedures are described. |
| Q5 — Analytical coverage | Unclear | The transition from 1,958 participants with ABPM/MRI to 1,734 analyzed is described, including clinical and missing-data exclusions. Coverage of the entire cohort and its subgroups remains uncertain. |
| Q6 — Condition identification | Yes | ABPM and explicit thresholds are reported. INH additionally requires no history of antihypertensive medication; the published 18.1% should not be labeled prevalence among all untreated participants. |
| Q7 — Measurement consistency | Yes | A common device, daytime 30-minute and nighttime hourly readings, >70% of expected total measurements, and minima of 20 daytime and seven nighttime readings are specified. |
| Q8 — Statistical analysis | No | The report gives 314 INH cases among 1,734 participants and descriptive percentages, but no confidence interval for this prevalence. The reported confidence intervals describe adjusted means or associations, not INH frequency. The review-calculated Wilson interval does not change this source-level reporting limitation. The mixed-treatment denominator and the no-medication-history case definition remain explicitly distinguished. |
| Q9 — Response and non-response | Unclear | Information is insufficient to evaluate non-response to the ABPM/MRI substudy against all eligible parent-cohort participants. |

Applicability, assessed separately: ABPM/MRI community subset; 527 of 1,734 on medication, while INH requires no medication history. Current treatment and treatment history are not equivalent.

Table S8. GRADE-informed certainty judgments for the retained pooled proportions.

Certainty was assessed using a GRADE-informed approach adapted to prevalence [29,30]. Appropriate prevalence designs started at high certainty; outcome-specific reasons for downgrading are presented below.

| **Domain** | **NH** | **Study-defined INH** | **Basis / interpretation** |
| --- | --- | --- | --- |
| Risk of bias | Serious | Serious | Selection into ABPM, coverage, and non-response are recurrent concerns. Uncertainty in the IDACO extraction is noted in Table S4a. Missing source-level prevalence confidence intervals were recorded in Q8; no additional downgrade was applied after the review calculated those intervals. |
| Inconsistency | Very serious | Very serious | NH: I² 97.6%, PI 17.2–72.3%. INH: I² 91.5%, PI 3.6–54.0%. Large unexplained variation is judged from the whole distribution and protocols, not I² alone. |
| Indirectness | Serious | Serious | Age/treatment restrictions limit extrapolation to unrestricted adults. INH additionally uses different treatment denominators and history rules. Geographic diversity alone is not a downgrade. |
| Imprecision | CI 33.2–52.3%; no additional threshold-based downgrade | CI 10.4–27.5%; no additional threshold-based downgrade | No prespecified screening decision threshold permits a universal binary precision rule. Width is reported explicitly; absence of an extra downgrade does not mean that precision is adequate for a clinical decision. |
| Reporting bias | Uncertain; no additional automatic downgrade | Uncertain; no additional automatic downgrade | No formal asymmetry test is informative with four independent sources. Lack of testing is not evidence of no bias. Search and selection limitations are described directly. |
| Overall certainty | Very low | Very low | Both outcomes remain very low after the stated risk-of-bias, inconsistency and indirectness judgments. No certainty rating is provided for a withdrawn clinical/circadian pooled estimate. |

No summed JBI score, D5–D8 rule, or automatic confidence-interval-width threshold was used.

Table S9a. Analytical-component mapping and source-level sensitivity.

| **Estimate identifier** | **Source identifier** | **Cases / N** | **Independence / inclusion decision** |
| --- | --- | --- | --- |
| IDACO_Copenhagen_2017 | IDACO [1] | 859/2,142 | One of six IDACO analytical components; common original publication. |
| IDACO_Ohasama_2017 | IDACO [1] | 368/1,275 | One of six IDACO analytical components; common original publication. |
| IDACO_Noorderkempen_2017 | IDACO [1] | 233/943 | One of six IDACO analytical components; common original publication. |
| IDACO_Montevideo_2017 | IDACO [1] | 415/962 | One of six IDACO analytical components; common original publication. |
| IDACO_EPOGH_2017 | IDACO [1] | 191/491 | One of six IDACO analytical components; common original publication. Aggregates Novosibirsk, Pilsen, Padua and Kraków (four cohorts). |
| IDACO_Maracaibo_2017 | IDACO [1] | 346/544 | One of six IDACO analytical components; common original publication. |
| Li_2007_JingNing_total_NH | Li [2] | 334/677 | Independent source publication Primary JingNing report; IDACO JingNing omitted. |
| Ogedegbe_2013_JHS_total_NH | Ogedegbe [3] | 233/425 | Independent source publication |
| Rhee_2021_Korea_total_NH_untreated | Rhee [4] | 352/823 | Independent source publication |

The IDACO publication includes 6,546 participants; exclusion of 189 JingNing participants leaves 6,357. Its six components contribute 2,412 extracted NH cases. EPOGH combines four cohorts.

The two-stage source-level analysis yielded 47.3% (95% CI, 37.1–57.8; approximate prediction interval, 22.2–73.8%). Omitting IDACO yielded 48.8% (95% CI, 34.3–63.5; k=3).

Table S9b. Interpretation of contextual and non-extractable original reports.

| **Report** | **Report type** | **Current role** | **Why it does not define a pooled population estimate** |
| --- | --- | --- | --- |
| Omboni/TEMPLAR 2019 [6] | Community-pharmacy registry | Contextual / not pooled | Pharmacy-referred ABPM; INH count is approximate. |
| Etyang/Kenya 2016 [7] | Original population diagnostic study | Contextual / not pooled | Population ABPM, but no separable nighttime numerator for the prioritized outcome. |
| Melgarejo/Maracaibo 2022 [8] | Original population/MRI sub-sample | Contextual / not pooled | Population-derived MRI subset using ≥110/65; not combined with 120/70. |
| Amjo/Nigeria 2020 [9] | Original clinical study in hypertensive patients | Contextual / not pooled | Referred hypertensive clinical sample, not a population prevalence denominator. |
| de la Sierra/Spain ABPM 2014 [10] | Clinical registry | Contextual / not pooled | Clinical registry; systolic-only ≥120 and dipping. Treatment strata displayed separately. |
| Barochiner/Buenos Aires 2023 [11] | Original tertiary hospital study | Contextual / not pooled | Tertiary-hospital clinical sampling; individual proportions only. |
| Banegas/MUCH 2014 [12] | Treated clinical registry | Contextual / not pooled | Masked uncontrolled hypertension conditional on treated/clinic-controlled status. |
| Minetto/La Plata 2026 [13] | Original outpatient clinical study | Contextual / not pooled | Cardiometabolic outpatients; referral-based population. |
| Chuwa/Tanzania 2020 [14] | Original occupational study | Contextual / not pooled | University employees; occupational recruitment. |
| Huang/Shanghai 2022 [15] | Nested case-control/source clinical population | Contextual / not pooled | Clinic source population 155/1,368; no use of case-control denominator. |
| Kaul/India ABPM 2019 [16] | Clinical all-comers registry | Contextual / not pooled | Clinically referred registry; circadian phenotypes only. |
| Kario/JAMP 2020 [17] | High-risk clinical cohort | Contextual / not pooled | High-risk clinical cohort; individual NH/circadian proportions only. |

Contextual reports are not an exhaustive evidence base for their respective populations or outcomes.

Supplementary references

1. Melgarejo JD, Maestre GE, Thijs L, et al. Prevalence, treatment, and control rates of conventional and ambulatory hypertension across 10 populations in 3 continents. Hypertension. 2017;70:50-58.

2. Li Y, Staessen JA, Lu L, Li LH, Wang GL, Wang JG. Is isolated nocturnal hypertension a novel clinical entity? Findings from a Chinese population study. Hypertension. 2007;50:333–339.

3. Ogedegbe G, Spruill TM, Sarpong DF, et al. Correlates of isolated nocturnal hypertension and target organ damage in a population-based cohort of African Americans: the Jackson Heart Study. Am J Hypertens. 2013;26:1011–1016.

4. Rhee MY, Kim JS, Kim CH, et al. Prevalence and characteristics of isolated nocturnal hypertension in the general population. Korean J Intern Med. 2021;36:1126–1133.

5. Kim SH, Shin C, Kim S, et al. Prevalence of isolated nocturnal hypertension and development of arterial stiffness, left ventricular hypertrophy, and silent cerebrovascular lesions: the Korean Genome and Epidemiology Study. J Am Heart Assoc. 2022;11:e025641.

6. Omboni S, Mancinelli A, Rizzi F, Parati G; TEMPLAR Project Group. Telemonitoring of 24-hour blood pressure in local pharmacies and blood pressure control in the community: the TEMPLAR Project. Am J Hypertens. 2019;32(7):629–639. doi:10.1093/ajh/hpz049.

7. Etyang AO, Warne B, Kapesa S, et al. Clinical and epidemiological implications of 24-hour ambulatory blood pressure monitoring for the diagnosis of hypertension in Kenyan adults: a population-based study. J Am Heart Assoc. 2016;5:e004797.

8. Melgarejo JD, Maestre GE, Gutierrez J, et al. Subclinical magnetic resonance imaging markers of cerebral small vessel disease in relation to office and ambulatory blood pressure measurements. Front Neurol. 2022;13:908260.

9. Amjo I, Adebayo RA, Akinyele OA, et al. Diurnal rhythm of blood pressure among Nigerians with hypertension using 24-hour ambulatory blood pressure monitoring. Pan Afr Med J. 2020;36:240.

10. de la Sierra A, Gorostidi M, Banegas JR, Segura J, de la Cruz JJ, Ruilope LM. Nocturnal hypertension or nondipping: which is better associated with the cardiovascular risk profile? Am J Hypertens. 2014;27:680-687.

11. Barochiner J, Díaz RR, Martínez R. Prevalence and characteristics of isolated nocturnal hypertension and masked nocturnal hypertension in a tertiary hospital in the city of Buenos Aires. Diagnostics. 2023;13:1419.

12. Banegas JR, Ruilope LM, de la Sierra A, et al. High prevalence of masked uncontrolled hypertension in people with treated hypertension. Eur Heart J. 2014;35(46):3304–3312. doi:10.1093/eurheartj/ehu016.

13. Minetto J, Espeche W, Cerri G, et al. Isolated nocturnal hypertension and its association with sleep duration and quality. J Hum Hypertens. 2026;40:96-102.

14. Chuwa G, Chillo P. Ambulatory blood pressure profiles and correlation with cardiovascular risk factors in a sample of 390 university employees in Tanzania. Integr Blood Press Control. 2020;13:197-208.

15. Huang JF, Zhang DY, Sheng CS, et al. Isolated nocturnal hypertension in relation to host and environmental factors and clock genes. J Clin Hypertens. 2022;24:1255-1262.

16. Kaul U, Omboni S, Arambam P, et al. Blood pressure related to age: the India ABPM study. J Clin Hypertens. 2019;21:1784-1794.

17. Kario K, Hoshide S, Mizuno H, et al. Nighttime blood pressure phenotype and cardiovascular prognosis: practitioner-based nationwide JAMP Study. Circulation. 2020;142:1810–1820.

18. Staplin N, de la Sierra A, Ruilope LM, et al. Relationship between clinic and ambulatory blood pressure and mortality: an observational cohort study in 59 124 patients. Lancet. 2023;401:2041–2050.

19. Doyle CY, Ruiz JM, Taylor DJ, et al. Associations between objective sleep and ambulatory blood pressure in a community sample. Psychosom Med. 2019;81:545-556.

20. Parati G, Pengo MF, Avolio A, et al. Nocturnal blood pressure: pathophysiology, measurement and clinical implications. Position paper of the European Society of Hypertension. J Hypertens. 2025;43:1296–1318.

21. Liu J, Li Y, Zhang X, et al. Management of nocturnal hypertension: an expert consensus document from Chinese Hypertension League. J Clin Hypertens. 2024;26:71–83.

22. Cuspidi C, Sala C, Tadic M, et al. Clinical and prognostic significance of a reverse dipping pattern on ambulatory monitoring: an updated review. J Clin Hypertens. 2017;19:713-721.

23. Kim SH, Kim YH, Kim JS, et al. Target-organ damage and incident hypertension: the Korean Genome and Epidemiology Study. J Hypertens. 2016;34:524-531.

24. Röver C, Knapp G, Friede T. Hartung-Knapp-Sidik-Jonkman approach and its modification for random-effects meta-analysis with few studies. BMC Med Res Methodol. 2015;15:99. doi:10.1186/s12874-015-0091-1.

25. Viechtbauer W. Meta-analysis via linear (mixed-effects) models: rma.uni. metafor documentation [Internet]. [cited 2026 Sep 17]. Available from: https://wviechtb.github.io/metafor/reference/rma.uni.html

26. Röver C, Friede T. Double arcsine transform not appropriate for meta-analysis. Res Synth Methods. 2022;13(5):645–648. doi:10.1002/jrsm.1591.

27. Joanna Briggs Institute. Critical appraisal checklist for studies reporting prevalence data. Adelaide: Joanna Briggs Institute; 2017 [cited 2026 Sep 17]. Available from: https://jbi.global/sites/default/files/2019-05/JBI_Critical_Appraisal-Checklist_for_Prevalence_Studies2017_0.pdf

28. Munn Z, Moola S, Lisy K, Riitano D, Tufanaru C. Methodological guidance for systematic reviews of observational epidemiological studies reporting prevalence and cumulative incidence data. Int J Evid Based Healthc. 2015;13(3):147–153. doi:10.1097/XEB.0000000000000054.

29. Iorio A, Spencer FA, Falavigna M, et al. Use of GRADE for assessment of evidence about prognosis: rating confidence in estimates of event rates in broad categories of patients. BMJ. 2015;350:h870. doi:10.1136/bmj.h870.

30. Borges Migliavaca C, Stein C, Colpani V, Barker TH, Munn Z, Falavigna M; PERSyst Group. How are systematic reviews of prevalence conducted? A methodological study. BMC Med Res Methodol. 2020;20:96. doi:10.1186/s12874-020-00975-3.

Figure S1. Two-stage NH sensitivity with IDACO represented by one source estimate.

The IDACO row is the first-stage pooled estimate, not its crude case fraction. Cases/N identify source size. The second stage conditions on the estimated first-stage variance; intervals are approximate. Squares are uniform and do not show weights.

Figure S2. INH sensitivity using reported non-current-treatment denominators.

JingNing uses 606 and KoGES 1,207 participants. Source-specific treatment-history definitions and age restrictions remain. The diamond and lower line are the modified Hartung–Knapp CI and approximate PI.

Figure S3. Individual NH observations in clinical, occupational and high-risk reports.

Non-exhaustive contextual inventory. No pooled estimator, summary diamond or between-study weights.

Figure S4. Individual contextual INH observations.

TEMPLAR’s approximate count and its conditional Wilson interval are marked with ≈. These are not estimates from one common clinical target population.

Figure S5. Individual contextual non-dipping observations.

Phenotype labels are retained from the previous extraction. Definitions and sampling frames are not assumed to be equivalent; there is no pooled estimate.

Figure S6. Individual contextual reverse/riser observations.

These observations do not form an exhaustive outcome-specific review. No pooled prevalence is shown.
